# Root spatial metabolite profiling of two genotypes of barley *(Hordeum vulgare* L.) reveals differences in response to short-term salt stress

**DOI:** 10.1093/jxb/erw059

**Published:** 2016-03-05

**Authors:** Megan C. Shelden, Daniel A. Dias, Nirupama S. Jayasinghe, Antony Bacic, Ute Roessner

**Affiliations:** ^1^Australian Centre for Plant Functional Genomics, School of Agriculture, Food and Wine, University of Adelaide, Glen Osmond SA 5064, Australia; ^2^Metabolomics Australia, The University of Melbourne, Parkville VIC 3010, Australia; ^3^ARC Centre of Excellence in Plant Cell Walls, School of BioSciences, The University of Melbourne, Parkville VIC 3010, Australia; ^4^School of BioSciences, The University of Melbourne, Parkville VIC 3010, Australia

**Keywords:** Cell division, cereal, gas chromatography–mass spectrophotometry, metabolomics, osmotic stress, root elongation, root growth, salinity.

## Abstract

Barley (*Hordeum vulgare* L.) is the most salt-tolerant cereal crop and has excellent genetic and genomic resources. It is therefore a good model to study salt-tolerance mechanisms in cereals. We aimed to determine metabolic differences between a cultivated barley, Clipper (tolerant), and a North African landrace, Sahara (susceptible), previously shown to have contrasting root growth phenotypes in response to the early phase of salinity stress. GC-MS was used to determine spatial changes in primary metabolites in barley roots in response to salt stress, by profiling three different regions of the root: root cap/cell division zone (R1), elongation zone (R2), and maturation zone (R3). We identified 76 known metabolites, including 29 amino acids and amines, 20 organic acids and fatty acids, and 19 sugars and sugar phosphates. The maintenance of cell division and root elongation in Clipper in response to short-term salt stress was associated with the synthesis and accumulation of amino acids (i.e. proline), sugars (maltose, sucrose, xylose), and organic acids (gluconate, shikimate), indicating a potential role for these metabolic pathways in salt tolerance and the maintenance of root elongation. The processes involved in root growth adaptation and the underlying coordination of metabolic pathways appear to be controlled in a region-specific manner. This study highlights the importance of utilizing spatial profiling and will provide us with a better understanding of abiotic stress response(s) in plants at the tissue and cellular level.

## Highlight

The maintenance of cell division and root elongation in barley appears to be associated with the synthesis of specific metabolites, indicating a potential role for these in salt tolerance.

## Introduction

Salt-affected soil is a major agricultural problem, with over 800 million ha of land estimated to be affected by salinity worldwide and 32 million ha of dryland agriculture estimated to be salt affected (FAO, 2015). Important cereal crops such as barley (*Hordeum vulgare*) and wheat (*Triticum* spp.) are growing in increasingly hostile environments, resulting in significant decreases in yield. The effect of salinity on plant growth has previously been described by a two-phase model (Munns, 2002). The early response to salt stress is referred to as an osmotic stress but is more accurately described as a shoot ion-accumulation independent stress as it occurs within minutes and continues over the duration of the salt stress (Roy *et al.*, 2014). Osmotic stress is a result of low water potential around the root and results in cell dehydration; loss of cell turgor; and inhibition of cell division, cell expansion, and photosynthesis; leading to a reduction in plant growth and yield (Munns and Tester, 2008). In contrast, ionic stress is a result of the accumulation of toxic concentrations of Na^+^ and Cl^−^ in the cytoplasm of shoot tissues and usually occurs after long-term exposure to salt. High concentrations of Na^+^ have been shown to accumulate in root cortical cells and cell walls, resulting in a decrease in cell turgor and root growth (Flowers and Hajibagheri, 2001), indicating that the roots may also elicit a salt-specific response.

Salinity has been shown to reduce root growth in many plant species, including *Arabidopsis* (Wu *et al.*, 1996), *Gossypium hirsutum* (cotton; Zhong and Lauchli, 1993), and the cereals *Oryza sativa* (rice; Lin and Kao, 1996), barley (Shelden *et al.*, 2013), wheat (Rahnama *et al.*, 2011), and *Zea mays* (maize; Bernstein and Kafkafi, 2002). Some plants maintain root elongation at salt concentrations (or in other low water potential media) that inhibit shoot growth (Spollen *et al.*, 1993; Yamaguchi and Sharp, 2010); this is an important adaptive mechanism ensuring seedling establishment and allowing an increase in soil root exploration for water and nutrient uptake (Yamaguchi and Sharp, 2010). In a study of eight genotypes of barley, increasing salinity resulted in a progressive reduction in seminal root elongation rate (Shelden *et al.*, 2013). Considerable variation in root elongation was observed among the genotypes, with wild barley identified as the most tolerant. A reduction in root growth in response to salt stress is often associated with an inhibition of cell division and cell expansion (Chatterjee and Majumder, 2010), as has been shown previously in barley (Tabur and Demir, 2009, 2010). Understanding the response of the root apical meristem, and of elongation and differentiation processes to environmental stresses will be important for understanding tolerance mechanisms in the root.

The response of roots to salinity is often transient; within hours, turgor can be restored by osmotic adjustment (Pritchard *et al.*, 1991). Osmotic adjustment occurs through the synthesis and accumulation of osmoprotectants (compatible solutes) and inorganic ions (Na^+^, K^+^, and Cl^−^). In barley genotypes, root and shoot ion concentrations do not correlate with root elongation rates, suggesting Na^+^ and K^+^ concentrations are not directly influencing root growth (Shelden *et al.*, 2013). In the elongation zone of the maize primary root, osmotic adjustment occurs at low water potentials through an increase in proline deposition due to an increase in proline transport into the root tip (Voetberg and Sharp, 1991).

Recent advances in high-throughput functional genomics technologies make it possible to gain more insight into salt-tolerance mechanisms. Metabolism is likely to vary between tissues and cell types and these changes can often go undetected when bulk tissues are analysed. Thus, there is a need to use both spatial and temporal resolution to elucidate the molecular response to salinity (and other abiotic stresses). With the advances in analytical technologies it is now possible to measure metabolite changes in tissues or single cells (Wang and Bodovitz, 2010; Shelden and Roessner, 2013). In Arabidopsis, this technology has been used to produce a high-resolution metabolite map of specific cell types in the root using fluorescence-activated cell sorting (Moussaieff *et al.*, 2013). The metabolite responses to abiotic stress have been studied extensively in plants (Urano *et al.*, 2010; Obata and Fernie, 2012; Shelden and Roessner, 2013). The response to salinity has been studied in a number of agriculturally important plants, including barley (Widodo *et al.*, 2009; Wu *et al.*, 2013) and *Vitis vinifera* (grapevine; Cramer *et al.*, 2007); however, these studies were all conducted on bulk tissues (i.e. whole shoots or roots) and thus it is likely that some metabolites present in either specific tissues or cell types would have been undetected.

**Fig. 1.**
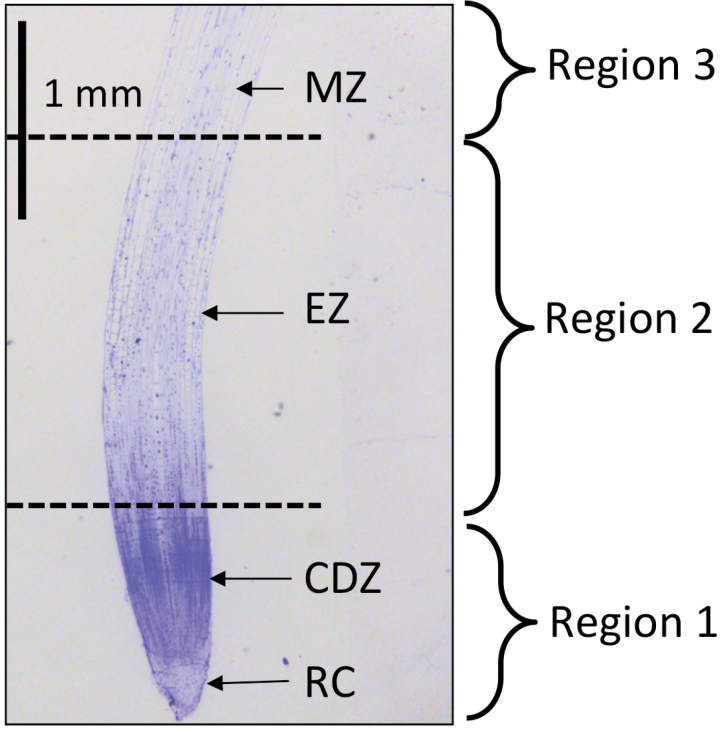
Longitudinal section through a 2-day-old barley cv. Clipper seminal root tip stained with toluidine blue. CDZ, cell division zone; EZ, elongation zone; MZ, maturation zone; RC, root cap.

In terms of production, barley is the fourth most important cereal crop worldwide (after maize, rice, and wheat) producing 144 MT in 2013 (http://faostat3.fao.org/browse/rankings/commodities_by_regions/E) (FAO, 2015). It is more salt tolerant than other glycophytic cereals, such as wheat, making it a good model for studying salt-tolerance mechanisms. For this study, we used two barley genotypes, Clipper (malting barley) and Sahara (landrace), known to have contrasting salinity-tolerance mechanisms (Widodo *et al.*, 2009; Shelden *et al.*, 2013). Clipper (tolerant) has previously been shown to maintain root elongation in response to salt stress, whilst in Sahara (sensitive) root elongation was significantly reduced (Shelden *et al.*, 2013). In order to determine spatial changes in primary metabolites involved in osmotic adjustment in barley seminal roots in response to salt stress, we profiled primary metabolites involved in central metabolism in three different regions of the root: the root cap/cell division zone (R1), elongation zone (R2), and maturation zone (R3) (Fig. 1). A GC-MS metabolite profiling approach was used to monitor the metabolic changes between the different regions of the root and in response to short-term salt stress. To our knowledge, this is the first study to use spatially resolved metabolomics to gain insight into the short-term root response to salt stress.

## Materials and methods

### Plant material and growth conditions

*Hordeum vulgare* L. cv. Clipper (Australian malting variety) and the landrace Sahara (North Africa) were used for all experiments. Barley seeds were sourced from the Australian Centre for Plant Functional Genomics (Adelaide, South Australia). Growth conditions were as described previously (Shelden *et al.*, 2013). Briefly, seeds were germinated and transferred to nutrient agar medium for 48h. For salinity treatment, nutrient agar was supplemented with 100mM NaCl and additional CaCl_2_ to a final activity of 1mM (Genc *et al.*, 2007; Shelden *et al.*, 2013). Seedlings were harvested for microscopy and metabolomics after 48h growth on agar media. Over this time period, seminal root growth has previously been shown to be linear and lateral roots are not yet formed (Shelden *et al.*, 2013).

### Chemical fixation

Seminal roots of barley seedlings cv. Clipper and Sahara (n = 5) were harvested after 48h growth on agar media. A surgical blade was used to excise 4mm root tips from the root cap junction, which were then immediately immersed in 2.5% glutaraldehyde in 1× phosphate buffered saline (PBS; pH 7.4), and stored at 4°C. Excised root tips were fixed in 2.5% glutaraldehyde in PBS (pH 7.4) for 2h at room temperature. The samples were rinsed three times in fresh buffer for 10min each, before being dehydrated in increasing concentrations of ethanol consisting of 10, 30, 50, 70, 90, and 100% anhydrous ethanol for 30min at each step. Following dehydration the samples were infiltrated with increasing concentrations of LR white resin (Sigma) in ethanol consisting of 25, 50, 75, and 100% resin for 8h at each step. After a second change of 100% resin the tissues were embedded in fresh resin in gelatine capsules. The gelatine capsules were capped to exclude air and the resin polymerised in an oven at 60°C for 24h.

### Light microscopy

Embedded root tips in resin blocks were sectioned longitudinally with a diamond knife on a Leica Ultracut-S microtome. Semi-thin sections (500nm) were collected onto glass slides and dried on a warm hotplate. Sections were stained with alkaline toluidine blue for 5min before being gently rinsed in a stream of distilled water until all the excess stain was removed. The stained sections on slides were dried and viewed with a DM2500 Leica compound microscope and digital images captured with a Leica 300F digital camera. Average cell lengths were calculated by measuring the length of 10–15 cortical cells at various positions from the root apex.

### Sampling of 2-day-old roots for metabolomics

The apical region of the root was cut into three sections measured from the root cap junction (Fig. 1). R1 comprised the root cap and cell division zone, R2 the elongation zone, and R3 the maturation zone (defined as where the cortical cell length was uniform). Sections from the three longest seminal roots of 10 individual seedlings were pooled for each biological representative (n = 5). In total, 200 plants were used and the fresh weight (FW) for pooled samples ranged from 1.8 to 18.3mg. Owing to instrument sensitivity issues, we pooled tissue from each root region from multiple plants to ensure we had sufficient material to detect and identify as many metabolites as possible. The length of the root sections varied according to cultivar and treatment differences: Clipper (control) R1 0–1mm, R2 1–3.5mm, and R3 3.5–6.0mm; Clipper (100mM NaCl) R1 0–1.0mm, R2 1.0–3.0mm, and R3 3.0–5.0mm; Sahara (control) R1 0–1.0mM, R2 1.0–3.0mm, and R3 3.0–5.0mm; Sahara (100mM NaCl) R1 0–0.5mm, R2 0.5–2.5mm, and R3 2.5–5.0mm. Root sections were immediately transferred to aluminium boats that were floating in liquid N_2_. The pooled root sections were immediately weighed to prevent thawing to determine the FW of each sample and subsequently snap frozen in 2mL Eppendorf tubes and stored at −80°C.

### Extraction and derivatization

Approximately 10mg of the various sections of root tissue (R1, R2, and R3) was added to a Cryomill tube. Next, 150 μL methanol (100%) with 2 µL internal standards (^13^C_6_-sorbitol/^13^C_5_^15^N-valine in water, 0.2mg mL^−1^) was added. The sample was subsequently homogenized using a Cryomill (Bertin Technologies) using programme #2 (6100–3×45×45) at −10°C. The sample mixture was vortexed for 30s and then incubated for 15min at 70°C at 850rpm. Milli-Q water (150 μL) was added and then the sample was centrifuged at 13000rpm for 5min. A 90 μL aliquot was transferred into a glass insert and dried *in vacuo* for subsequent trimethylsilyl (TMS) and tri-*tert*-butyldimethylsilyl (TBS) polar metabolite derivatization.

### Polar metabolite TMS and TBS derivatization

The dried samples were re-dissolved in 10 μL of 30mg mL^−1^ methoxyamine hydrochloride in pyridine and derivatized at 37°C for 120min with mixing at 500rpm using the on-line derivatization capability of the GERSTEL autosampler. One dried aliquot was then treated for 30min with 20 μL *N*,*O*-*bis*-(trimethylsilyl)trifluoroacetamide (BSTFA) and another for 45min with 20 μL *N-(tert*-butyldimethylsilyl*)-N*-methyltrifluoroacetamide (MTBSTFA). To both samples, 2.0 μL retention time standard mixture [0.029% (*v*/*v*) *n* dodecane, *n*-pentadecane, *n*-nonadecane, *n*-docosane, *n*-octacosane, *n*-dotriacontane, *n*-hexatriacontane dissolved in pyridine] was added, with mixing at 500rpm at 37°C (TMS) or 45°C (TBS). Each derivatized sample was allowed to rest for 60min prior to injection.

### GC-MS analysis

The GC-MS analysis was conducted as described previously (Jacobs *et al.*, 2007; Hill *et al.*, 2013). For the polar TMS metabolite analysis, the temperature programme described in Hill *et al.* (2013) was used. For the TBS metabolite analysis, the following temperature programme was used: start injection at 100°C, hold for 1min, then a 12.5°C min^−1^ oven temperature increase to 325°C, with a final 6min of heating at 325°C. Both chromatograms and mass spectra were evaluated using the AnalyzerPro Deconvolution programme (Spectralworks, UK). Mass spectra of eluting compounds were identified using the public domain mass spectra library of Max Planck Institute for Plant Physiology, Golm, Germany (http://csbdb.mpimp-golm.mpg.de/csbdb/dbma/msri.html) and the in-house Metabolomics Australia mass spectral library. All matching mass spectra were additionally verified by determining the retention time by analysis of authentic standard substances. Resulting relative response ratios (area of analyte divided by area of internal standard, ^13^C_6_-sorbitol for TMS-derivatized metabolites and/or ^13^C_5_^15^N-valine for TBS-derivatized metabolites) per sample FW (mg) for each analysed metabolite as described in Roessner *et al.* (2001). If a specific metabolite had multiple TMS or TBS derivatives, the metabolite with the greater detector response and improved peak shape within the dynamic range of the instrument was selected.

### Statistical analysis

The metabolite data were analysed as described in Roessner *et al.* (2001) and are presented as fold changes relative to a reference that is set to 1. Statistical analysis of metabolite data was performed using Microsoft Excel. Differences between samples were validated by the Student’s *t*-test incorporated in Excel. Differences were considered significant with a *t*-test value *P* < 0.05 (Bonferroni-corrected *P* value) and between the Bonferroni corrected value and 0.05. The heatmap was generated with the open-source software, MetaboAnalyst 3.0 (http://www.metaboanalyst.ca/MetaboAnalyst/faces/home.xhtml).

## Results

### The root growth response and cellular anatomy is altered in response to salt

Previously we have shown a differential response in the root elongation of two cultivars of barley, Clipper and Sahara, to short-term salt stress (Shelden *et al.*, 2013). In order to further evaluate this difference, we examined the cortical cell length in the seminal roots to determine if the effects of 100mM NaCl on root elongation were due to an inhibition of cell expansion and/or cell division (Table 1). In the more salt-tolerant Clipper, the length of the cell division region was approximately 1mm and appeared to be unaffected by the addition of salt. In the elongation zone, cortical cells had variable lengths and thus were assumed to be undergoing cell expansion. In control plants, the elongation zone in Clipper was approximately 2mm in length. In a previous study we showed that in response to salt, growth rate was reduced by 20% compared to control plants (Shelden *et al.*, 2013). In this study we have shown the cortical cell length becomes uniform closer to the root apex, thus the elongation zone is shortened by salt treatment. The start of the maturation zone was defined as where the cortical cell length became uniform. Final cortical cell length was similar for both control and salt-treated Clipper roots (Table 1). In the more salt-sensitive Sahara, the cells started expanding closer to the root apex and the length of the elongation zone was unaltered in response to salt, most likely indicating an inhibition of cell division (Table 1). It was also observed that in response to salt treatment the final cortical cell length in Sahara roots was double that of control plants (Table 1).

**Table 1.** Spatial distribution of cortical cell length in Clipper and Sahara roots. The effect of 48h treatment with 100mM NaCl on the spatial distribution of cortical cell length in the apical 5mm of Clipper and Sahara seminal roots was examined compared with untreated roots.

**Genotype**	**Clipper**		**Sahara**	
**Treatment**	**Control**	**Salt**	**Control**	**Salt**
Region 1 (mm)	0–1.0	0–1.0	0–1.0	0–0.5
Region 2 (mm)	1.0 – 3.5	1.0–3.0	1.0–3.0	0.5–2.5
Region 3 (mm)	3.5–5.5	3.0 – 5.0	3.0–5.0	2.5–4.5
Final cortical cell length (μm)	141.9±8.5^a^	143.4±11.6^a^	108.8±3.9^a^	220.5±15.0^b^

The length of each region was defined by the following: region 1, root cap and cortical cells dividing; region 2, cortical cells expanding; and region 3, cortical cells ceased expansion and the length became uniform. The final cortical cell length shown is the mean ± SEM (n = 8–10). Different superscript letters indicate significance between the salt treatment relative to control and between genotypes (two-way ANOVA, Bonferroni post-hoc test, *P* < 0.001).

### Metabolite response differs spatially in the root

In order to precisely measure metabolic changes in the different regions of barley seminal roots, and in response to salt treatment, the roots were sectioned according to the estimates obtained from the cortical cell length measurements (Table 1). We employed GC-MS to measure a wide range of metabolites. In total, 98 metabolites were measured; of these, 76 were identified unambiguously and included 29 amino acids (AA) and amines, 20 organic acids (OA), and fatty acids (FA), and 19 sugars and sugar phosphates. Of those only the metabolites that changed significantly are further discussed below (for a complete list of metabolites see Supplementary Tables S1, S2, and S3). We found that metabolite profiles changed spatially along the root and were different between the two genotypes and in response to salt stress.

### Spatial metabolic profiles in the seminal roots of Clipper and Sahara

**Fig. 2.**
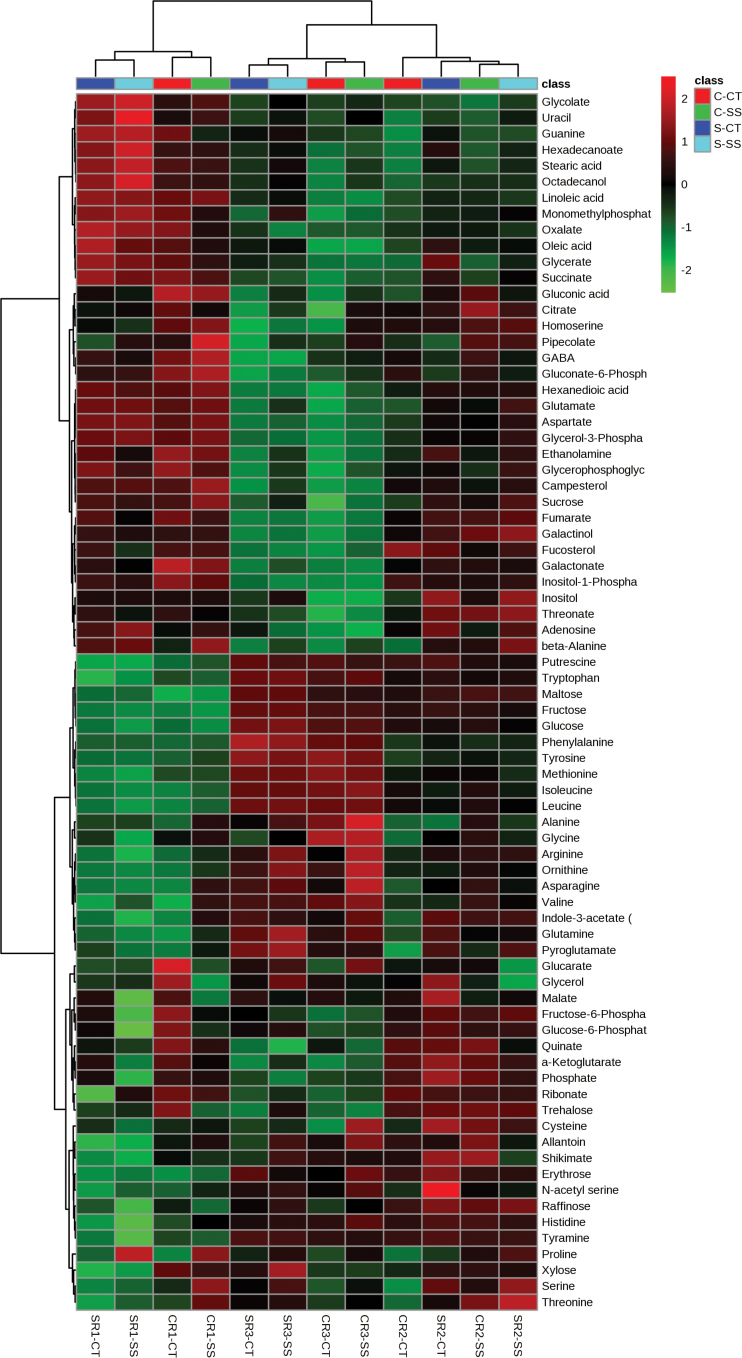
Clustered heatmap of the normalized metabolite log response between root zones/treatment and the measured metabolites in Clipper and Sahara. Clustering of the roots zones and salt treatments is depicted by the dendrogram at the top. Clustering of the metabolites is depicted by the dendrogram at the left. Each coloured cell (red higher, green lower) on the map corresponds to a normalized log response value of the metabolite levels, with samples in columns and metabolites in rows. CRl CT, Clipper R1 Control; SR1 CT, Sahara R1 Control; CR1 SS, Clipper R1 salt-stressed; SR1 SS, Sahara R1 salt-stressed; CR2 CT, Clipper R2 Control; SR2 CT, Sahara R2 Control; CR2 SS, Clipper R2 salt-stressed; SR2 SS, Sahara R2 salt-stressed; CR3 CT, Clipper R3 control; SR3 CT, Sahara R3 control; CR3 SS, Clipper R3 salt-stressed; SR3 SS, Sahara R3 salt-stressed.

**Fig. 3.**
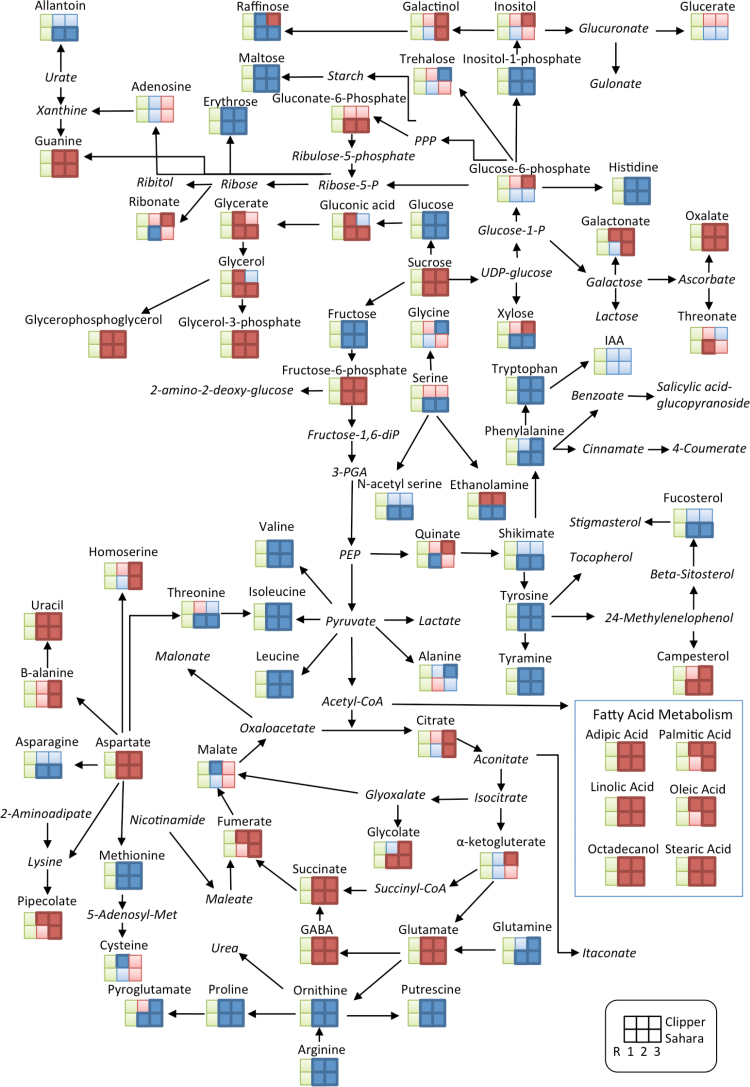
Analysis of metabolites in the root development zones (R1, R2, and R3) in barley genotypes Clipper and Sahara grown in control conditions. The identified metabolites are mapped onto a simplified metabolic network. Fold changes were determined for each root zone (R2, elongation zone; R3, maturation zone) relative to R1, cell division zone (pale green squares). Dark blue (dark red) squares indicate a significant fold-increase (decrease) in R2/R3 compared to R1. Light blue (light red) squares indicate an increase (decrease) in R2/R3 compared to R1 but these are not significant. Metabolite names in italics represent metabolites not detected in this study.

The heatmap analysis shows a clear separation between the three regions of the root (R1, R2, and R3) for both Clipper and Sahara (Fig. 2). The majority of metabolites detected changed significantly along the developmental gradient of the root in both genotypes (Fig. 3, Supplementary Table S1 and S2). Significant fold-changes were described relative to R1 (Fig. 1). In Clipper roots, there were significant spatial changes to OAs, FAs, sugars, and sugar phosphates along the root (Fig. 3, Supplementary Table S1). The general trend for AAs in Clipper was an increase in their relative response ratio, with levels highest in R3 and lowest in R1. The following AAs and amines increased significantly in R2 and/or R3 compared to R1: valine, isoleucine, leucine, alanine, tyrosine, phenylalanine, tryptophan, arginine, methionine, ornithine, putrescine, tyramine, histidine, glycine, proline, pyroglutamate, and glutamine. The largest increases in AAs were putrescine (R3; 12-fold), tyramine (R2; 6-fold), and tryptophan (R3; 9-fold) (Supplementary Table S1). Conversely, the AAs aspartate, **γ**-aminobutyric acid (GABA), glutamate, homo-serine, and β-alanine, and the amines ethanolamine and guanine decreased with cell maturity, with levels highest in R1. Components of the tricarboxylic acid (TCA) cycle (citrate, succinate, fumarate, and α-ketoglutarate) were highest in the most apical region of the root tip (R1) and decreased shootward with levels lowest in R3 (Fig. 3, Supplementary Table S2). Components of lipid metabolism (adipic acid, palmitic acid, octadecanol, linoleic acid, stearic acid, and oleic acid) were also highest in R1. With the exception of shikimate, all other OA significantly decreased when compared to R1. The sugars maltose (51-fold, 29-fold), fructose (27-fold, 54-fold), glucose (10-fold, 23-fold), and erythrose (4.3-fold, 3-fold) increased in R2 and R3 respectively, when compared with R1. Raffinose increased only in R2 compared with R1 (2.5-fold). In R3, the largest significant increases were in fructose (54-fold) and glucose (23-fold). All other sugars measured decreased significantly relative to R1.

**Fig. 4.**
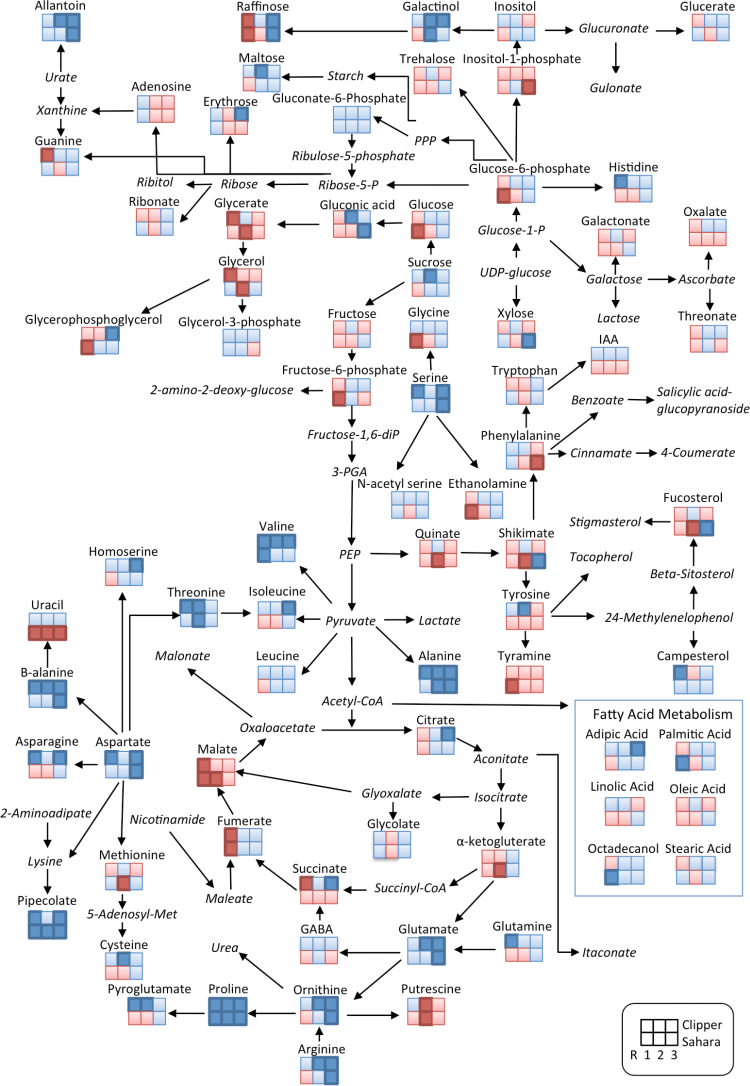
Analysis of metabolites in the root development zones (R1, R2, and R3) in barley genotypes Clipper and Sahara grown on 100mM salt. The identified metabolites are mapped onto a simplified metabolic network. Fold changes were determined for 100mM salt treatment compared to control for each root zone. Dark blue (dark red) squares indicate a significant fold-increase (decrease) in response to salt treatment compared to control conditions. Light blue (light red) squares indicate an increase (decrease) in response to salt treatment compared to control conditions but these are not significant. Metabolite names in italics were not detected in this study.

In Sahara, the relative response ratios of AAs generally increased spatially along the root towards the shoot (Fig. 4). The following AAs and amines increased significantly in R2 and/or R3 compared to R1: putrescine, tryptophan, tyramine, histidine, allantoin, threonine, *N*-acetyl serine, methionine, glutamine, arginine, leucine, asparagine, isoleucine, ornithine, phenylalanine, tyrosine, serine, pyroglutamate, valine, and proline (Supplementary Table S2). The largest fold changes in R2 and R3, respectively, were putrescine (25-fold, 31-fold), tryptophan (16-fold, 47-fold), tyramine (16-fold, 14-fold), and histidine (6.6-fold, 4.4-fold). The AAs β-alanine, glutamate, aspartate, guanine, GABA, and amine ethanolamine significantly decreased from R1 towards R3. The relative responses of measured components involved in FA metabolism (adipic acid, hexadeconoate, octadecanol, linoleic acid, and oleic acid) were highest in R1 and decreased shootwards towards R3, as seen in Clipper roots. Components of the TCA cycle (citrate, succinate, glycolate, and fumarate) were highest in the most apical region of the root tip (R1) and decreased shootwards with levels lowest in R3. The sugars fructose, glucose, and xylose increased significantly and were highest in R3, while raffinose, xylose, erythrose, ribonate, and glycerol were highest in R2.

Although we identified many similarities in the metabolic response of Clipper and Sahara roots in control conditions, some important differences between genotypes were also observed (Fig. 3, Supplementary Tables S1 and S2). The sugars xylose, raffinose, ribonate, glucose-6-phosphate, galactonate, and inositol all had a differential response between genotypes. A number of AAs also differed, including asparagine, glycine, serine, threonine, and ethanolamine, and the OAs quinate and galactonate.

### Metabolic changes in response to short-term salt stress

To determine the response of each genotype to salinity stress, we compared the level of each metabolite in each region of the root with the same metabolite measured in the same region of the control plants. The metabolic response was different for each genotype and in each region of the root. The heatmap analysis shows that for R1 and R3 there was clear separation between genotypes and salt treatment; however, in R2 the response was more variable and we did not observe a clear separation between genotypes and salt treatment (Fig. 2).

### Metabolic changes in response to salt stress in Clipper roots

In response to salt treatment, there was a general trend seen in the roots of Clipper with increases in most AAs compared to the controls (Fig. 4, Supplementary Table S3). The AAs alanine, proline, asparagine, β-alanine, and valine were significantly increased in all three regions of the root. In R1, there were significant increases in asparagine (3.8-fold), threonine (2.3-fold), alanine (1.8-fold), histidine (1.8-fold), glutamine (1.7-fold), serine (1.6-fold), isoleucine (1.3-fold), aspartate (1.3-fold), pyroglutamate (1.3-fold), and leucine (1.2-fold) (Supplementary Table S3). Significant decreases in response to salt in R1 included the amine guanine (0.4-fold); OAs glycerate (0.8-fold), succinate (0.8-fold), fumarate (0.6-fold), and malate (0.5-fold); and the sugars raffinose (0.4-fold) and glycerol (0.3-fold).

**Fig. 5.**
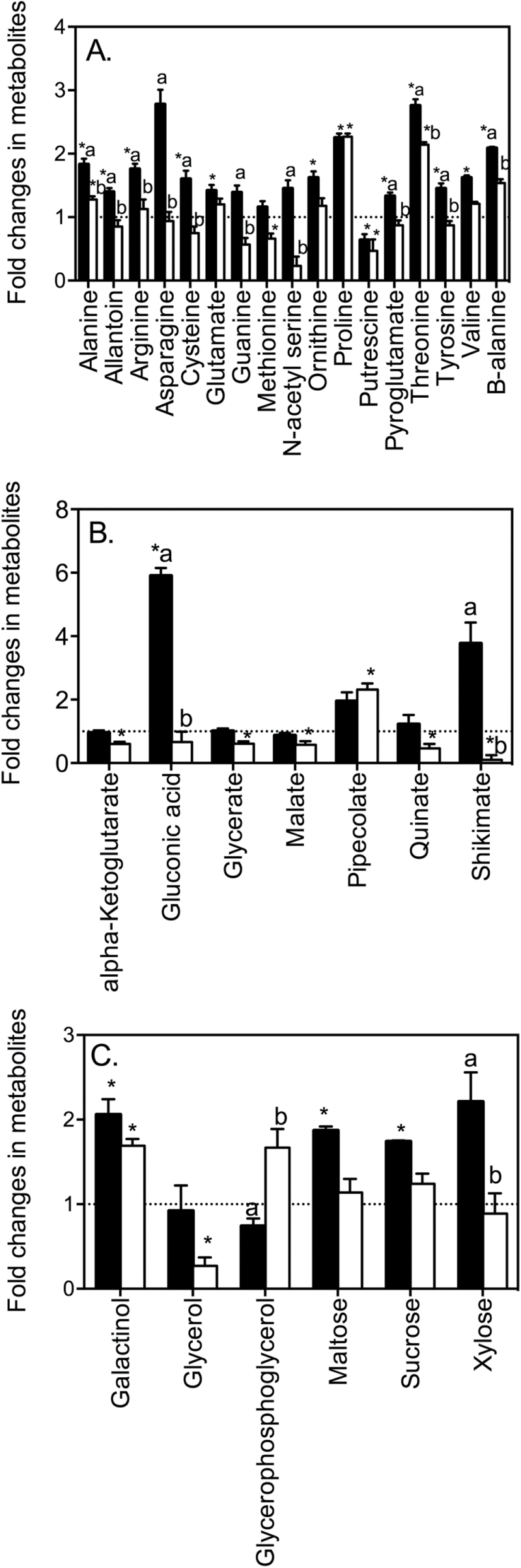
Comparison of metabolite changes in the elongation zone (R2) of Clipper and Sahara in response to 48h salt stress. Fold-changes for Clipper (black bars) and Sahara (white bars) were determined relative to the control for **A** amino acids, **B** organic acids, and **C** sugars. Only metabolites that changed significantly are shown (see Supplementary Table S3 for all data). * denotes metabolites that are significantly different to a fold-change of 1 (student’s *t*-test). Superscript letters indicate significant difference between genotypes Clipper and Sahara (two-way ANOVA, *P* < 0.01).

In R2 (elongation zone), there was a general increase in AAs, sugars, sugar phosphates, and OAs in response to salt treatment in Clipper roots (Fig. 5, Supplementary Table S3). Threonine (2.8-fold), proline (2.3-fold), β-alanine (2.1-fold), alanine (1.8-fold), allantoin (1.4-fold), glutamate (1.4-fold), arginine (1.8-fold), cysteine (1.6-fold), ornithine (1.6-fold), valine (1.6-fold), pyroglutamate (1.3-fold), tyrosine (1.5-fold), and the sugars galactinol (2-fold), maltose (1.9-fold), and sucrose (1.7-fold) all significantly increased compared to the control (Fig. 5A, B and C). The most significant increase in response to salt was the OA gluconate (5.9-fold) (Fig. 5B). There was only one metabolite, putrescine (0.6-fold), that significantly decreased in R2.

In R3 (maturation zone) of Clipper roots, there were small increases (up to 2-fold) in the following AAs and amines: allantoin, proline, alanine, β-alanine, glutamate, homo-serine, aspartate, isoleucine, serine, and valine (Fig. 4, Supplementary Table S3). Asparagine (3.5-fold), arginine (2.9-fold), and orthinine (2.7-fold) also increased in response to salt stress. The sugars erythrose (2.2-fold), glycerophosphoglycerol (2.5-fold), raffinose (1.8-fold), and galactinol (1.4-fold) increased significantly. The OAs citrate (2.1-fold), pipecolate (1.7-fold), and succinate (1.3-fold) also increased, as did the FA hexanedioic acid (1.8-fold). R3 in Clipper showed no significant decreases in metabolites in response to salt.

### Metabolic changes in response to salt stress in Sahara roots

In R1, the AAs proline (5.4-fold) and valine (1.5-fold) increased and glycine (0.8-fold), arginine (0.6-fold), and the amine tyramine (0.3-fold) decreased (Fig. 4, Supplementary Table S3). The FAs octadecanol (1.7-fold) and hexadecanoate (1.5-fold) increased and the OAs fumarate (0.5-fold) and malate (0.5-fold) decreased, as did the sugars fructose-6-phosphate (0.3-fold), glucose 0.5-fold), glucose-6-phosphate (0.3-fold), and raffinose (0.3-fold). Campesterol and monomethylphosphate increased (1.7-fold) and ethanolamine (0.7-fold), fucosterol (0.5-fold), and uracil (0.4-fold) decreased.

In R2, the overall trend in AAs, OAs, and FAs in response to salt was a decrease (Fig. 5). The AAs proline (2.3-fold), threonine (2.1-fold), and alanine (1.3-fold) significantly increased and methionine (0.7-fold) and the amine putrescine (0.5-fold) decreased (Fig. 5A). Pipecolate increased by 2.3-fold and glycerate (0.6-fold), α-ketoglutarate (0.6-fold), malate (0.6-fold), phosphate (0.6-fold), quinate (0.5-fold), shikimate (0.1-fold), and uracil (0.6-fold) all significantly decreased in response to salt (Fig. 5B). The sugar alcohols galactinol (1.7-fold) increased and glycerol (0.3-fold) decreased (Fig. 5C).

In R3, there were significant increases in many metabolites in response to salt. The AAs and amines allantoin, ornithine, arginine, proline, β-alanine, glutamate, alanine, aspartate, and serine all increased significantly in response to salt, whilst phenylalanine decreased (0.8-fold). The OAs shikimate (3.9-fold), gluconic acid (2.5-fold), monomethylphosphate (2.2-fold), and pipecolate (1.8-fold) increased and phosphate decreased (0.8-fold). The sugars xylose (3.2-fold) and raffinose (1.6-fold) increased, while inositol-1-phosphate (0.8-fold) and uracil (0.8-fold) decreased.

### Differential metabolite accumulation in the root zones of Clipper and Sahara

**Fig. 6.**
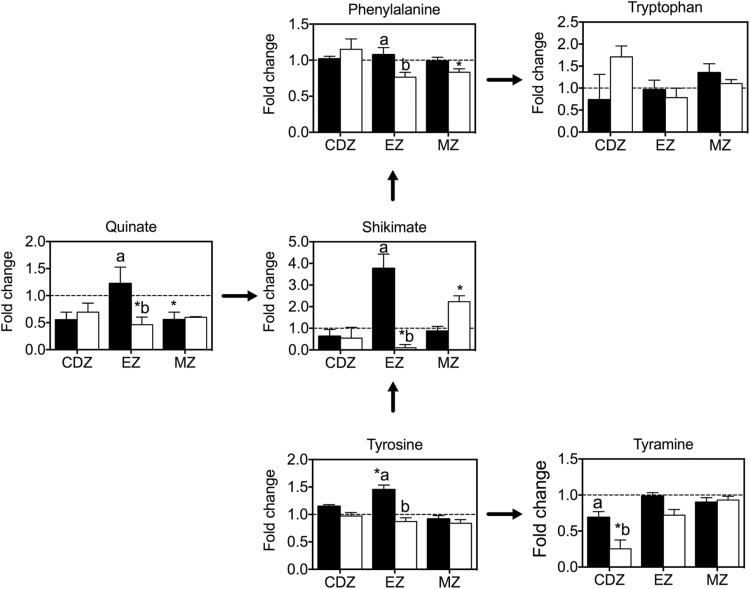
Changes in metabolites in the shikimate pathway of Clipper and Sahara in response to 48h salt stress. Fold-changes for Clipper (black bars) and Sahara (white bars) were determined relative to the control. * denotes metabolites that are significantly different to a fold-change of 1 (student’s *t*-test). Superscript letters indicate significant difference between genotypes Clipper and Sahara (two-way ANOVA, *P* < 0.01).

In response to short-term salt stress, there was a general decrease in OAs (specifically components of the TCA cycle) and sugars in R1 and an increase in AAs in R3 in both Clipper and Sahara roots (Fig. 4, Supplementary Table S3). In both genotypes the known osmoprotectant proline increased in response to salt stress, with the highest accumulation in R1 (Fig. 4, Supplementary Table S3). The most pronounced differences in metabolite profiles between the two genotypes were in the elongation zone (R2), where there was a significant increase in some AAs, OAs, and sugars in Clipper and a decrease in these metabolites in Sahara (Fig. 5). Alanine, allantoin, arginine, asparagine, cysteine, guanine, N-acetyl serine, pyroglutamate, threonine, tyrosine, and β-alanine were all significantly higher in the elongation zone of Clipper than in Sahara (Fig. 5A). The fold-changes in gluconate, shikimate, and xylose relative to the control were significantly higher in the elongation zone of Clipper roots than Sahara roots (Fig. 5B, C). In Clipper, metabolites in the shikimate pathway were significantly increased in the elongation zone (quinate, shikimate, phenylalanine, and tyrosine) and cell division zone (tyramine) (Fig. 6) compared to Sahara. Salt stress resulted in a significant decrease in uracil in all root zones of Sahara compared with no significant change in Clipper.

## Discussion

Here we have shown the importance of performing metabolite analysis on specific regions of the root as a tool for understanding the changes in the growth zone. We have also shown that genotypes with contrasting root-growth response to short-term salt stress have different metabolite profiles. By carrying out GC-MS–based metabolomics we were able to assign specific metabolite changes to specific regions of the root. A plants’ response to environmental stress occurs at all levels of organization and thus it is important to study the stress response at a cellular and tissue level. In a recent study in *Medicago truncatula,* a transcriptomics and metabolomics approach was used to gain insight into the metabolic differences between root tips and border cells (Watson *et al.*, 2015). Here we describe the first detailed metabolite profiling in different spatial regions of the barley root. The results indicate that the metabolome in the barley root is region and genotype specific as well as salt responsive.

In the root tip of the primary root, new cells are generated from cell division in the apical meristem for both the root cap and cells that will undergo further cell division, expansion, and differentiation. The process of cell division in the root is highly regulated both spatially and temporally (Benfey *et al.*, 2010). As the cells migrate away from the root tip and become fully expanded they undergo the complex process of cell differentiation into the epidermis, cortex, endodermis, and vasculature of the primary root.

Genetic variation in root phenotypes have previously been reported in barley (Bengough *et al.*, 2004) and wheat (Richards *et al.*, 2007; Wasson *et al.*, 2014) and in response to abiotic stresses (James *et al.*, 2008; Shavrukov *et al.*, 2010; Shelden *et al.*, 2013). We have previously shown variation in seminal root elongation in eight genetically diverse genotypes of barley (Shelden *et al.*, 2013). The root phenotype of Clipper is inherently different from the landrace Sahara, with Clipper having longer roots. The root phenotypes of Clipper and Sahara are altered in response to abiotic stresses including boron toxicity and salinity (Roessner *et al.*, 2006; Choi *et al.*, 2007; Widodo *et al.*, 2009; Shelden *et al.*, 2013). In order to gain a molecular understanding of the differences in root elongation in these two genotypes, we performed metabolite analyses by dividing the root tips into three segments corresponding with the apical meristem/cell division zone, elongation zone, and maturation zone (Fig. 1).

The metabolite analysis indicated that there were region-specific responses corresponding to root development (Figs 2 and 3). Both Clipper and Sahara showed very similar trends in their metabolic response along the developmental gradient under control conditions; however, there were some notable differences. Higher levels of TCA cycle intermediates in the cell division zone in both genotypes most likely indicate the increased energy requirement for cell division (Supplementary Tables S1 and S2). FAs are a major component of membranes and were highest in the cell division zone where cell membrane synthesis is occurring. AAs increased along the developmental gradient towards the shoot, contributing to an increase in protein synthesis involved in cell elongation, maturation, and differentiation (Fig. 6).

The growth of the root system depends on the availability of carbon to the roots (Muller *et al.*, 1998). Cell division and cell expansion are thought to be two independent processes (Green, 1976), but both are affected by carbon availability. Cell division can be inhibited by carbon depletion (Muller *et al.*, 1998; Van’t Hof, 1968). The process of cell elongation not only requires expansion of the cell wall but also an accumulation of intracellular solutes, including sugars, to generate an osmotic potential and facilitate water uptake into the cell (Cosgrove, 1999). Gradients in soluble sugars, including sucrose, glucose, and fructose, were seen along the root tip, with sucrose highest at the apex and glucose and fructose increasing further away from the apex and into the maturation zone. These trends are similar to what has been reported previously in maize (Sharp *et al.*, 1990; Muller *et al.*, 1998). This may be a result of either the termination of phloem in the maturation zone or the absence of sucrose-cleaving enzymes (invertases) in the root apex (Hellebust and Forward, 1962). Significant differences in both glucose and xylose were observed between Clipper and Sahara and potentially indicate differences in cell wall composition between the cultivated barley and the landrace; however, this needs to be evaluated directly by measuring cell wall composition and nucleotide sugars. In rice, genotype differences in glucose and xylose were shown to correlate with specific hemicellulosic polymers (Zhang *et al.*, 2012). The accumulation of the raffinose family oligosaccharides inositol, galactinol, and raffinose also showed differences between the two genotypes. These are important storage compounds and osmoprotectants and may reflect differences in response and adaptation to environmental stresses between cultivated barley and the landrace Sahara.

Interestingly, GABA showed a distinct profile in both genotypes, with higher levels in the cell division zone. GABA is believed to play an important role in many processes, including maintaining the carbon/nitrogen balance, pH regulation, signalling, and energy production (Fait *et al.*, 2008; Ramesh *et al.*, 2015), and has been shown to accumulate in response to both biotic and abiotic stresses (Kinnersley and Turano, 2000). In Arabidopsis, *pop2* mutants, which have a reduced ability to degrade GABA, accumulate high levels of GABA that result in an inhibition of cell elongation and represses cell wall synthesis genes (Renault *et al.*, 2011). The role of GABA in plant development is not well studied (Renault *et al.*, 2011) and to our knowledge there is no direct evidence of a role for GABA in root cell division.

The shikimate pathway (Fig. 6) is involved in biosynthesis of aromatic AAs, including tryptophan, tyrosine, and phenylalanine, and these serve as precursors for a wide range of secondary metabolites including lignin (Herrmann and Weaver, 1999; Maeda and Dudareva, 2012). Quinate synthesis branches from the main trunk of the shikimate pathway and is an ubiquitous plant building block for phytoalexins and UV protectants (Herrmann and Weaver, 1999). In plants, 20% of carbon is fixed through the shikimate pathway and this is linked to the biosynthesis of aromatic compounds (Herrmann, 1995). Increases in the aromatic AAs in both genotypes in the elongation zone and maturation zone are most likely owing to the differentiation of cells into specialized cell types requiring the synthesis of secondary metabolites and structural compounds, including lignin and suberin (Fan *et al.*, 2006; Barros *et al.*, 2015). The observed differences in the accumulation of quinate and shikimate in Clipper and Sahara roots may be a result of differences in the regulation of these pathways (Fig. 6).

### Metabolite response to short-term salt stress in the roots

The ability of seedlings to maintain root elongation in saline soils is an important adaptation for seedling establishment and ensuring an adequate water and nutrient supply for shoot growth. The cellular response to salt stress can include changes in the cell cycle and division, and modifications to cellular membranes and cell walls (Shabala and Munns, 2012). Root elongation is often inhibited by osmotic stress and this can be due to an inhibition of cell division and/or cell expansion (Munns and Tester, 2008). The anatomical study demonstrated that the susceptible genotype Sahara has a significant inhibition in the cell division that is primarily responsible for a decrease in root elongation rate in response to salinity. Interestingly, cortical cell length in the root increased dramatically in response to salinity in Sahara roots. Cell enlargement in response to salinity has been reported previously in maize (Li *et al.*, 2014) and halophytes (Hajibagheri *et al.*, 1985) and may be a result of increased vacuolation—an adaptive mechanism to cope with increased intracellular ion concentration.

One mechanism to cope with osmotic stress is the sequestering of inorganic ions (Na^+^ and Cl^−^) into the vacuole. In response to abiotic stress, plants can also alter their metabolism and can synthesize compatible solutes (i.e., proline, glycine betaine, sucrose, and raffinose) to cope with the changes in water potential and maintain turgor pressure. Compatible solutes accumulate in the cytosol and organelles to help facilitate osmotic adjustment; however, the synthesis of compatible solutes is more energetically expensive than sequestering ions into the vacuole and thus this comes at a cost to the plant (Shabala and Munns, 2012). The concentrations of compatible solutes that accumulate are thought to be in the order of 10mM but can be higher when partitioned into the cytoplasm (Munns and Tester, 2008). This study provides novel insights into the metabolic response of barley seedlings exposed to salt stress by using spatial metabolite profiling of the root.

**Fig. 7.**
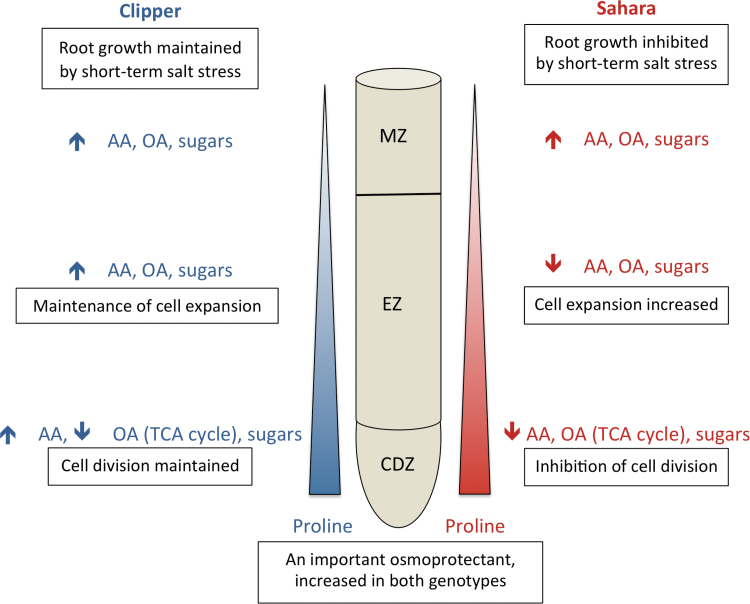
Schematic diagram showing the major trends in metabolic changes of AAs, OAs, and sugars in the maturation zone (MZ), elongation zone (EZ), and cell division zone (CDZ) of Clipper (blue) and Sahara (red) roots in response to short-term salt stress. Upward arrows indicate an increase in response to salt stress and downward arrows indicate a decrease in response to salt stress.

Salt-tolerant glycophytes (e.g. barley) can help mitigate the effects of salt stress by the synthesis of compatible solutes. Proline has been shown to act as an osmoprotectant in plants affected by both drought (Bowne *et al.*, 2012) and salinity (Ueda *et al.*, 2007). Increases in proline in response to salt stress have previously been reported in both barley roots and leaves (Widodo *et al.*, 2009; Wu *et al.*, 2013); however, proline levels do not always correlate with salt tolerance (Chen *et al.*, 2007). Interestingly, both Clipper and Sahara had similar increases in proline accumulation in response to salt stress, with proline levels highest in the most apical region of the root where cell division was occurring (Fig. 7, Supplementary Table S3). Increased accumulation of proline in the apical region of the root tip (with values up to 160mM measured at the root apex) has previously been observed in maize in response to water stress (Voetberg and Sharp, 1991; Verslues and Sharp, 1999; Raymond and Smirnoff, 2002; Yamaguchi and Sharp, 2010) and is thought to contribute significantly to osmotic adjustment. In Arabidopsis, *P5CS* (Δ1-Pyrroline-5-carboxylase synthase), which regulates proline accumulation, has been shown to be induced by moderate salt stress, thus providing osmotic stress tolerance (Liu and Zhu, 1997). It been suggested that proline may also play a role in cell division in Arabidopsis (Mattioli *et al.*, 2009).

In Clipper, there were significant increases in AAs, OAs, and sugars in all three regions of the root, most likely contributing to the ability of Clipper roots to maintain elongation in response to salt stress (Fig. 7). Interestingly, the elongation zone of Clipper showed the most contrasting response compared to Sahara (Fig. 5) with significant increases in some AAs and amines (alanine, allantoin, asparagine, cysteine, guanine, N-acetyl serine, pyroglutamate, threonine, tyrosine, and β-alanine), sugars (xylose), and OAs (gluconate and shikimate). This corresponds with the ability of Clipper to maintain cell expansion (Table 1) and root elongation rate (Shelden *et al.*, 2013). Gluconate has previously been shown to increase in response to salt stress in barley roots (Wu *et al.*, 2013). Glucose dehydrogenase catalyses the oxidation of glucose to gluconate and this enzyme has been implicated in conferring tolerance to plant cells in response to stress (Witzel *et al.*, 2010).

Nitrogen-containing compounds (alanine, arginine, leucine, ornithine, serine, and valine), the amides (asparagine and glutamine), and polyamines (putrescine and spermine) have been shown to accumulate as part of the stress response in plants (Rabe, 1990). In two rice genotypes with different salt tolerance levels, most AAs and sugars significantly increased in the leaves and roots of both genotypes; however, the salt-tolerant genotype had a more prolonged response over time (Zhao *et al.*, 2014). Asparagine and glutamine have both been reported to accumulate in response to salt stress (Imamul Huq and Larher, 1983; Mansour, 2000). Asparagine has a high N:C and thus is an ideal storage compound that accumulates under stress conditions (Lea *et al.*, 2007). Asparagine levels reportedly increased in the roots of barley seedlings (Yamaya and Matsumoto, 1989) and in barley leaves (Garthwaite *et al.*, 2005) in response to NaCl. In this study, asparagine increased in the root of the salt-tolerant Clipper in all three regions but not in the salt-sensitive Sahara and thus is likely involved in the ability of Clipper to maintain root elongation. Glutamine is the primary AA involved in nitrogen assimilation in plants and the amine group donor for many other compounds. Glutamine increased in response to salt stress in the cell division zone of Clipper, potentially indicating an increase in nitrogen assimilation. Uracil, a pyrimidine derivative, decreased in all root zones of Sahara, possibly reflecting changes in the biosynthesis or degradation of RNA in response to salinity (Zhang *et al.*, 2011).

Polyamines, including spermidine, spermine, and their precursor putrescine, are involved in plant growth and development and have been suggested to play an important role in plant stress responses (Gill and Tuteja, 2010). Putrescine is synthesized from arginine via either arginine decarboxylase or orthinine decarboxylase. In Clipper and Sahara, putrescine significantly decreased in response to salt in the elongation zone. Differences in the accumulation of putrescine in response to salt stress in rice are contradictory, with some studies reporting increases in salt-sensitive rice cultivars (Katiyar and Dubey, 1990) and other studies reporting an accumulation in roots of a tolerant variety compared to a sensitive variety (Lefèvre *et al.*, 2001). Widodo *et al.* (2009) reported an increase in putrescine after 5 weeks of salt stress in barley leaves associated with leaf senescence and cell damage. In *Vigna radiata* (mung bean), free putrescine decreased in roots in response to salt stress but increased in the leaves (Friedman *et al.*, 1989). The contradictory results of putrescine accumulation in response to salt stress may be a reflection of the tissue sampling procedures and further emphasize the need for spatial metabolomics analyses.

Components of the shikimate pathway including quinate, shikimate, phenylalanine, and tyrosine increased significantly in the elongation zone of Clipper in response to salt stress compared to Sahara (Figs 5 and 6). A significant decrease in shikimate and quinate in the elongation zone of Sahara roots (Fig. 6) indicated a salt-specific response in metabolite accumulation, as has been reported previously in Sahara (Widodo *et al.*, 2009). Thus, we have shown the shikimate salt response is region and genotype dependent, highlighting the importance of conducting spatial metabolite profiling. Because shikimate is the precursor to aromatic AAs, these results may indicate differences between the two genotypes in the synthesis of structural compounds such as lignin, which is involved in cell wall biosynthesis (Barros *et al.*, 2015). In the elongation zone of maize, up-regulation of cell wall-related genes was associated with cell enlargement in response to salinity (Li *et al.*, 2014).

In response to salt stress, sugar accumulation was highest in the elongation zone and is most likely involved in the maintenance of turgor pressure and carbohydrates for cell wall synthesis. In both Clipper and Sahara, sucrose levels were lowest in the root apex and glucose was highest in the maturation zone. This is the same as has been previously reported in maize (Sharp *et al.*, 1990; Muller *et al.*, 1998).

The structure and fluidity of plant membranes is crucial in a plant’s adaptation to abiotic stresses, and is affected by many factors including the FA composition of the lipids. Changes in the FA composition in response to salinity have been reported (Wu *et al.*, 1998; Chalbi *et al.*, 2013). In this study, FAs were found to be highest in R1 of the root, where cell division is occurring, most likely owing to the deposition of the cell membrane. The ratio of unsaturated to saturated FAs also altered in response to salt stress. In Sahara, an increase in the saturated FA palmitic acid in response to salt stress may be a mechanism to help restrict the uptake of Na^+^ into the plant root. Octadecanol is a long chain unsaturated fatty alcohol previously found in Arabidopsis epidermal cell surfaces (Franke *et al.*, 2005) and in the epicuticular wax of *Allium ampeloprasum* (leeks; Gabriela-Anca Maier and Post-Beittenmiller, 1998).

## Conclusion

We have used untargeted GC-MS metabolomics to identify differences along the development zones of the root in barley seedlings in response to salt stress. We have shown that the processes involved in root growth adaptation and the underlying coordination of metabolic pathways are controlled in a region-specific manner. In summary, the two genotypes had contrasting root growth responses to short-term salt stress and this was reflected in their metabolomes (Fig. 7). The synthesis of primary metabolites was spatially regulated in response to salt stress and differed between salt-tolerant and salt-sensitive genotypes. Clipper maintained cell division and root elongation through the synthesis of compatible solutes for osmotic adjustment and turgor maintenance. This study highlights the importance of utilizing ‘omics’ technologies for spatial profiling and provides us with potential metabolic pathways involved in root growth maintenance in response to salt stress. Further studies will involve matrix-assisted laser desorption/ionization imaging and transcriptional and genetic analyses to further elucidate the pathways involved in the root response.

## Supplementary data

Supplementary data are available at *JXB* online.

Table S1. Response ratios of metabolites in Region 2 and Region 3 compared to Region 1 in Clipper.

Table S2. Response ratios of metabolites in Region 2 and Region 3 compared to Region 1 in Sahara.

Table S3. Response ratios of metabolites in root zones of Clipper and Sahara subjected to salt stress.

Supplementary Data
