# Supplementary material for: Root spatial metabolite profiling of two genotypes of barley (Hordeum vulgare L.) reveals differences in response to short-term salt stress
Source: J Exp Bot. 2016 Mar 5;67(12):3731–45. doi: 10.1093/jxb/erw059 (PMC4896359; doi:10.1093/jxb/erw059)
Supplement: Supplementary Data [file supp_67_12_3731__index.html]

Root spatial metabolite profiling of two genotypes of barley (Hordeum vulgare L.) reveals differences in response to short-term salt stress — Root spatial metabolite profiling of two genotypes of barley (Hordeum vulgare L.) reveals differences in response to short-term salt stress — Supplementary Data 

# Root spatial metabolite profiling of two genotypes of barley *(Hordeum vulgare* L.) reveals differences in response to short-term salt stress

## Supplementary Data

Data files

- supplementary\_table\_S1\_S3.pdf - Supplementary Data
