# Supplementary material for: Root spatial metabolite profiling of two genotypes of barley (Hordeum vulgare L.) reveals differences in response to short-term salt stress
Source: J Exp Bot. 2016 Mar 5;67(12):3731–45. doi: 10.1093/jxb/erw059 (PMC4896359; doi:10.1093/jxb/erw059)
Supplement: Supplementary Data [file supp_erw059_supplementary_table_S1_S3.pdf]

Table S1: Response ratios of metabolites in Region 2 and Region 3 compared to Region 1 in Clipper

Metabolite	Region 1(CDZ)	Region 2 (EZ)	Region 3 (MZ)
**Amino acids and amines**			
Alanine	1.00 ± 0.09	1.05 ± 0.12	**2.66 ± 0.12**
Allantoin	1.00 ± 0.16	1.55 ± 0.11	1.21 ± 0.11
Arginine	1.00 ± 0.05	**1.52 ± 0.08**	**1.95 ± 0.14**
Asparagine	1.00 ± 0.35	1.41 ± 0.23	2.97 ± 0.09
Aspartate	1.00 ± 0.03	**0.28 ± 0.18**	**0.12 ± 0.02**
Butyric acid,4-amino (GABA)	1.00 ± 0.13	**0.36 ± 0.16**	**0.18 ± 0.17**
Cysteine	1.00 ± 0.09	1.00 ± 0.10	0.74 ± 0.17
Glutamate	1.00 ± 0.04	**0.42 ± 0.10**	**0.25 ± 0.05**
Glutamine	1.00 ± 0.04	1.47 ± 0.19	**2.56 ± 0.20**
Glycine	1.00 ± 0.05	0.86 ± 0.16	**1.56 ± 0.06**
Guanine	1.00 ± 0.35	0.18 ± 0.10	**0.28 ± 0.21**
Histidine	1.00 ± 0.14	**3.08 ± 0.16**	**2.97 ± 0.21**
Homoserine	1.00 ± 0.15	0.80 ± 0.08	**0.44 ± 0.10**
Isoleucine	1.00 ± 0.07	**3.30 ± 0.05**	**8.10 ± 0.04**
Leucine	1.00 ± 0.05	**2.92 ± 0.05**	**5.99 ± 0.03**
Methionine	1.00 ± 0.08	**1.50 ± 0.12**	**3.96 ± 0.07**
N-acetyl serine	1.00 ± 0.13	1.22 ± 0.18	1.79 ± 0.19
Ornithine	1.00 ± 0.14	**1.57 ± 0.08**	**3.39 ± 0.12**
Phenylalanine	1.00 ± 0.08	1.24 ± 0.07	**3.31 ± 0.07**
Proline	1.00 ± 0.12	1.29 ± 0.12	**1.61 ± 0.05**
Putrescine	1.00 ± 0.21	**9.18 ± 0.06**	**12.48 ± 0.11**
Pyroglutamate	1.00 ± 0.03	0.93 ± 0.06	**1.47 ± 0.07**
Serine	1.00 ± 0.06	0.88 ± 0.14	0.93 ± 0.04
Threonine	1.00 ± 0.34	0.94 ± 0.08	1.06 ± 0.16
Tryptophan	1.00 ± 0.15	**3.50 ± 0.24**	**9.12 ± 0.16**
Tyramine	1.00 ± 0.15	**5.86 ± 0.08**	**4.97 ± 0.06**
Tyrosine	1.00 ± 0.05	1.18 ± 0.07	**4.63 ± 0.09**
Valine	1.00 ± 0.20	**1.71 ± 0.06**	**3.25 ± 0.02**
β Alanine	1.00 ± 0.04	0.73 ± 0.15	**0.57 ± 0.05**
**Organic acids**			
alpha-Ketoglutarate	1.00 ± 0.13	1.07 ± 0.14	**0.37 ± 0.10**
Citrate	1.00 ± 0.14	0.77 ± 0.08	**0.38 ± 0.14**
Fumarate	1.00 ± 0.09	**0.47 ± 0.24**	**0.11 ± 0.19**
Glucarate	1.00 ± 0.20	0.60 ± 0.13	0.51 ± 0.22
Gluconic acid	1.00 ± 0.38	**0.07 ± 0.10**	**0.04 ± 0.41**
Glycerate	1.00 ± 0.06	**0.48 ± 0.06**	**0.46 ± 0.10**
Glycolate	1.00 ± 0.08	**0.59 ± 0.30**	**0.61 ± 0.11**
Malate	1.00 ± 0.07	0.81 ± 0.12	0.86 ± 0.08
Oxalate	1.00 ± 0.17	**0.48 ± 0.10**	**0.45 ± 0.27**
Pipecolate	1.00 ± 0.06	**0.65 ± 0.15**	**0.59 ± 0.04**
Quinate	1.00 ± 0.25	0.77 ± 0.13	**0.37 ± 0.20**
Shikimate	1.00 ± 0.22	1.51 ± 0.22	1.62 ± 0.15
Succinate	1.00 ±0 .07	**0.32 ± 0.08**	**0.23 ± 0.05**
Threonate	1.00 ± 0.09	0.92 ± 0.25	**0.42 ± 0.11**
**Fatty Acids**			
Palmitic Acid (Hexadecanoate)	1.00 ± 0.08	**0.32 ± 0.12**	**0.34 ± 0.11**
Adipic Acid (Hexanedioic acid)	1.00 ± 0.06	**0.42 ± 0.18**	**0.12 ± 0.21**
Stearic Acid (Octadecanoate)	1.00 ± 0.17	**0.22 ± 0.22**	**0.22 ± 0.12**
Linoleic acid	1.00 ± 0.30	**0.22 ± 0.16**	**0.15 ± 0.30**
Octadecanol	1.00 ± 0.18	**0.35 ± 0.22**	**0.29 ± 0.22**
Oleic acid	1.00 ± 0.18	**0.39 ± 0.20**	**0.22 ± 0.35**

**Sugars and Sugar Phosphates**			
Erythrose	1.00 ± 0.21	**4.28 ± 0.12**	**2.77 ± 0.24**
Fructose	1.00 ± 0.12	**27.70 ± 0.11**	**53.71 ± 0.09**
Fructose_6_Phosphate	1.00 ± 0.30	0.57 ± 0.12	**0.27 ± 0.18**
Galactinol	1.00 ± 0.08	0.78 ± 0.08	**0.22 ± 0.08**
Galactonate	1.00 ± 0.18	**0.26 ± 0.33**	**0.11 ± 0.09**
Gluconate-6-phosphate	1.00 ± 0.31	0.42 ± 0.21	0.11 ± 0.17
Glucose	1.00 ± 0.13	**9.73 ± 0.10**	**23.46 ± 0.09**
Glucose-6-phosphate	1.00 ± 0.32	0.67 ± 0.09	**0.35 ± 0.15**
Glycerol	1.00 ± 0.28	0.58 ± 0.12	0.63 ± 0.09
Glycerol-3-phosphate	1.00 ± 0.15	**0.23 ± 0.18**	**0.08 ± 0.13**
Glycerophosphoglycerol	1.00 ± 0.29	**0.20 ± 0.38**	**0.04 ± 0.09**
Inositol	1.00 ± 0.16	0.76 ± 0.12	**0.48 ± 0.12**
Inositol-1-phosphate	1.00 ± 0.23	0.64 ± 0.22	**0.17 ± 0.13**
Maltose	1.00 ± 0.24	**51.08 ± 0.57**	**28.71 ± 0.10**
Raffinose	1.00 ± 0.04	**2.45 ± 0.06**	**0.69 ± 0.09**
Ribonate	1.00 ± 0.19	0.87 ± 0.32	**0.16 ± 0.18**
Sucrose	1.00 ± 0.23	**0.39 ± 0.12**	**0.14 ± 0.19**
Trehalose	1.00 ± 0.20	0.88 ± 0.15	**0.57 ± 0.08**
Xylose	1.00 ± 0.38	0.29 ± 0.33	**0.24 ± 0.13**
**Other compounds**			
Adenosine	1.00 ± 0.29	1.06 ± 0.16	0.49 ± 0.35
Campersterol	1.00 ± 0.44	0.34 ± 0.15	**0.10 ± 0.08**
Ethanolamine	1.00 ± 0.13	**0.51 ± 0.15**	**0.27 ± 0.08**
Fucosterol	1.00 ± 0.15	1.39 ± 0.32	**0.31 ± 0.15**
Indole-3-acetate (IAA)	1.00 ± 0.18	1.21 ± 0.25	2.02 ± 0.28
Monomethylphosphate	1.00 ± 0.15	**0.39 ± 0.15**	**0.26 ± 0.39**
Phosphate	1.00 ± 0.08	1.10 ± 0.06	**0.55 ± 0.08**
Uracil	1.00 ± 0.11	**0.54 ± 0.16**	**0.64 ± 0.11**
**Unknowns**			
Un_11.54_417	1.00 ± 0.13	**0.34 ± 0.14**	**0.41 ± 0.17**
Un_12.91_273	1.00 ± 0.10	**2.95 ± 0.22**	**10.69 ± 0.08**
Un_13.96_284	1.00 ± 0.15	0.69 ± 0.07	0.74 ± 0.04
Un_14.37_158	1.00 ± 0.12	0.64 ± 0.20	**0.52 ± 0.18**
Un_14.87_191	1.00 ± 0.16	**0.42 ± 0.08**	**0.45 ± 0.16**
Un_15.01_115	1.00 ± 0.15	0.83 ± 0.07	1.28 ± 0.13
Un_15.07_261	1.00 ± 0.45	0.84 ± 0.17	0.86 ± 0.24
Un_16.77_591	1.00 ± 0.06	0.85 ± 0.15	**0.53 ± 0.09**
Un_17.26_102	1.00 ± 0.20	**3.15 ± 0.08**	1.49 ± 0.14
Un_18.1_345	1.00 ± 0.08	**1.71 ± 0.13**	**2.52 ± 0.05**
Un_18.11_116	1.00 ± 0.11	**0.31 ± 0.33**	**0.55 ± 0.15**
Un_18.4_242	1.00 ± 0.22	**0.34 ± 0.11**	**0.32 ± 0.16**
Un_19.41_156	1.00 ± 0.09	1.22 ± 0.10	**1.76 ± 0.14**
Un_22.25_217	1.00 ± 0.48	1.99 ± 0.05	**4.12 ± 0.09**
Un_25.61_379	1.00 ± 0.03	**2.37 ± 0.15**	**2.70 ± 0.21**
Un_26.96_433	1.00 ± 0.10	**3.50 ± 0.12**	**5.72 ± 0.23**
Un_27.2_217	1.00 ± 0.11	**0.52 ± 0.22**	**0.16 ± 0.09**
Un_28.39_446	1.00 ± 0.17	**4.82 ± 0.18**	**13.84 ± 0.22**
Un_28.68_446	1.00 ± 0.17	**4.94 ± 0.16**	**11.32 ± 0.18**
Un_30.54_217	1.00 ± 0.20	**12.15 ± 0.18**	**12.46 ± 0.09**
Un_30.72_217	1.00 ± 0.20	**12.84 ± 0.21**	**11.97 ± 0.08**
Un_33.11_433	1.00 ± 0.26	**0.47 ± 0.21**	**0.11 ± 0.10**

Data represents mean of 5 representatives of 10-pooled samples +/- s.e. Values that are highlighted in *green* (and bold) have a *t*-Test value $P < 0.05$ / (number of metabolites) while those with cyan (and bold) highlight are between this *P* value and 0.05 (i.e. below 0.05, but not below the Bonferroni-corrected *P* value).

Table S2: Response ratios of metabolites in Region 2 and Region 3 of Sahara compared to Region 1.

Metabolite	Region 1(CDZ)	Region 2 (EZ)	Region 3 (MZ)
**Amino acids and amines**			
Alanine	1.00 ± 0.10	0.82 ± 0.05	1.37 ± 0.11
Allantoin	1.00 ± 0.16	**2.93 ± 0.20**	**1.89 ± 0.04**
Arginine	1.00 ± 0.06	**2.46 ± 0.08**	**2.72 ± 0.11**
Asparagine	1.00 ± 0.05	**2.34 ± 0.12**	**4.00 ± 0.06**
Aspartate	1.00 ± 0.20	**0.40 ± 0.10**	**0.11 ± 0.04**
Butyric acid,4-amino (GABA)	1.00 ± 0.11	**0.34 ± 0.09**	**0.09 ± 0.30**
Cysteine	1.00 ± 0.03	**1.85 ± 0.14**	0.94 ± 0.15
Glutamate	1.00 ± 0.08	**0.66 ± 0.12**	**0.28 ± 0.09**
Ethanolamine	1.00 ± 0.07	0.89 ± 0.05	**0.36 ± 0.07**
Glutamine	1.00 ± 0.16	**2.46 ± 0.15**	**2.65 ± 0.15**
Glycine	1.00 ± 0.05	1.18 ± 0.07	0.95 ± 0.10
Guanine	1.00 ± 0.22	**0.35 ± 0.22**	**0.32 ± 0.21**
Histidine	1.00 ± 0.29	**6.62 ± 0.11**	**4.39 ± 0.11**
Homoserine	1.00 ± 0.14	1.17 ± 0.10	**0.57 ± 0.14**
Isoleucine	1.00 ± 0.10	**2.33 ± 0.04**	**6.57 ± 0.03**
Leucine	1.00 ± 0.07	**2.40 ± 0.05**	**5.64 ± 0.02**
Methionine	1.00 ± 0.12	**2.78 ± 0.09**	**5.17 ± 0.01**
N-acetyl serine	1.00 ± 0.25	**2.83 ± 0.15**	**3.12 ± 0.16**
Ornithine	1.00 ± 0.14	**2.16 ± 0.13**	**4.53 ± 0.19**
Phenylalanine	1.00 ± 0.08	**1.90 ± 0.14**	**5.30 ± 0.03**
Proline	1.00 ± 0.12	1.29 ± 0.11	**1.50 ± 0.02**
Putrescine	1.00 ± 0.27	**25.47 ± 0.16**	**31.37 ± 0.15**
Pyroglutamate	1.00 ± 0.14	**1.71 ± 0.14**	**1.64 ± 0.12**
Serine	1.00 ± 0.08	**1.87 ± 0.13**	**1.38 ± 0.04**
Threonine	1.00 ± 0.16	**2.89 ± 0.06**	**2.23 ± 0.11**
Tryptophan	1.00 ± 0.65	**16.76 ± 0.11**	**46.88 ± 0.09**
Tyramine	1.00 ± 0.32	**16.12 ± 0.13**	**14.41 ±0.09**
Tyrosine	1.00 ± 0.09	**1.89 ± 0.13**	**5.64 ± 0.03**
Valine	1.00 ± 0.11	**1.70 ± 0.11**	**2.76 ± 0.02**
β Alanine	1.00 ± 0.24	0.80 ± 0.20	**0.30 ± 0.07**
**Organic acids**			
alpha-Ketoglutarate	1.00 ± 0.29	1.84 ± 0.13	0.46 ± 0.26
Citrate	1.00 ± 0.09	1.19 ± 0.29	**0.64 ± 0.16**
Fumarate	1.00 ± 0.04	0.88 ± 0.17	**0.17 ± 0.14**
Glucarate	1.00 ± 0.30	1.26 ± 0.25	1.28 ± 0.16
Gluconic acid	1.00 ± 0.34	1.08 ± 0.25	**0.22 ± 0.25**
Glycerate	1.00 ± 0.05	0.88 ± 0.18	**0.54 ± 0.18**
Glycolate	1.00 ± 0.10	1.07 ± 0.52	**0.44 ± 0.09**
Malate	1.00 ± 0.02	1.63 ± 0.17	1.03 ± 0.10
Oxalate	1.00 ± 0.05	**0.45 ± 0.13**	**0.40 ± 0.08**
Pipecolate	1.00 ± 0.11	0.94 ± 0.08	**0.64 ± 0.10**
Quinate	1.00 ± 0.10	**2.26 ± 0.13**	0.51± 0.33
Shikimate	1.00 ± 0.44	**22.8 1 ± 0.45**	**4.33 ± 0.43**
Succinate	1.00 ± 0.07	**0.57 ± 0.07**	**0.30 ± 0.10**
Threonate	1.00 ± 0.18	1.43 ± 0.08	0.68 ± 0.20
**Fatty Acids**			
Palmitic Acid (Hexadecanoate)	1.00 ± 0.15	0.58 ± 0.27	**0.34 ± 0.11**
Adipic Acid (Hexanedioic acid)	1.00 ± 0.06	**0.50 ± 0.27**	**0.14 ± 0.06**
Stearic Acid (Octadecanoate)	1.00 ± 0.18	**0.33 ± 0.12**	**0.26 ± 0.12**
Linoleic acid	1.00 ± 0.19	**0.24 ± 0.39**	**0.22 ± 0.26**
Octadecanol	1.00 ± 0.08	**0.29 ± 0.28**	0.31 ± 0.11
Oleic acid	1.00 ± 0.20	**0.47 ± 0.11**	0.32 ± 0.38

**Sugars and Sugar Phosphates**			
Erythrose	1.00 ± 0.15	**7.83 ±0.14**	**5.78 ± 0.26**
Fructose	1.00 ± 0.26	**32.83 ± 0.33**	**73.93 ± 0.14**
Fructose_6_Phosphate	1.00 ± 0.14	1.45 ± 0.31	0.88 ± 0.11
Galactinol	1.00 ± 0.18	1.07 ± 0.02	**0.25 ± 0.14**
Galactonate	1.00 ± 0.10	1.05 ± 0.20	**0.30 ± 0.10**
Gluconate-6-phosphate	1.00 ± 0.14	**0.35 ± 0.20**	**0.13 ± 0.18**
Glucose	1.00 ± 0.13	**20.31 ± 0.55**	**43.97 ± 0.15**
Glucose-6-phosphate	1.00 ± 0.12	1.52 ± 0.29	1.02 ± 0.13
Glycerol	1.00 ± 0.16	**2.41 ± 0.26**	1.29 ± 0.07
Glycerol-3-phosphate	1.00 ± 0.09	**0.35 ± 0.14**	**0.11 ± 0.15**
Glycerophosphoglycerol	1.00 ± 0.03	**0.32 ± 0.18**	**0.07 ± 0.21**
Inositol	1.00 ± 0.11	6.57 ± 0.79	0.87 ± 0.11
Inositol-1-phosphate	1.00 ± 0.06	0.84 ± 0.26	**0.34 ± 0.04**
Maltose	1.00 ± 0.22	**12.13 ± 0.45**	**20.11 ± 0.07**
Raffinose	1.00 ± 0.22	**19.88 ± 0.55**	**2.60 ± 0.09**
Ribonate	1.00 ± 0.80	**2.55 ± 0.13**	0.68 ± 0.08
Sucrose	1.00 ± 0.10	0.85 ± 010	**0.31 ± 0.11**
Trehalose	1.00 ± 0.06	1.51 ± 0.18	0.86 ± 0.08
Xylose	1.00 ± 0.29	**9.18 ± 0.10**	**8.67 ± 0.27**
**Other compounds**			
Adenosine	1.00 ± 0.16	1.22 ± 0.28	0.63 ± 0.11
Campersterol	1.00 ± 0.12	**0.67 ± 0.09**	**0.18 ± 0.15**
Fucosterol	1.00 ± 0.07	**1.19 ± 0.12**	**0.67 ± 0.04**
Indole-3-acetate (IAA)	1.00 ± 0.37	2.65 ± 0.22	2.47 ± 0.24
Monomethylphosphate	1.00 ± 0.30	0.43 ± 0.19	**0.30 ± 0.13**
Phosphate	1.00 ± 0.12	**1.94 ± 0.06**	**0.67 ± 0.04**
Uracil	1.00 ± 0.08	**0.44 ± 0.17**	**0.45 ± 0.15**
**Unknowns**			
Un_11.54_417	1.00 ± 0.20	1.72 ± 0.75	**0.41 ± 0.22**
Un_12.91_273	1.00 ± 0.22	**2.52 ± 0.19**	**8.52 ± 0.44**
Un_13.96_284	1.00 ± 0.13	1.29 ± 0.12	1.11 ± 0.14
Un_14.37_158	1.00 ± 0.13	1.43 ± 0.14	0.93 ± 0.08
Un_14.87_191	1.00 ± 0.19	**0.41 ± 0.10**	**0.38 ± 0.10**
Un_15.01_115	1.00 ± 0.11	**1.71 ± 0.09**	**1.77 ± 0.13**
Un_15.07_261	1.00 ± 0.24	**2.84 ± 0.21**	2.10 ± 0.37
Un_16.77_591	1.00 ± 0.08	1.41 ± 0.20	0.68 ± 0.16
Un_17.26_102	1.00 ± 0.52	**14.34 ± 0.10**	**7.40 ± 0.21**
Un_18.1_345	1.00 ± 0.08	**2.86 ± 0.09**	**3.11 ± 0.07**
Un_18.11_116	1.00 ± 0.27	1.44 ± 0.30	**1.61 ± 0.11**
Un_18.4_242	1.00 ± 0.17	**0.52 ± 0.15**	**0.41 ± 0.07**
Un_19.41_156	1.00 ± 0.17	**2.59 ± 0.17**	**2.47 ± 0.15**
Un_22.25_217	1.00 ± 0.19	**6.52 ± 0.47**	**9.41 ± 0.10**
Un_25.61_379	1.00 ± 0.17	**3.41 ± 0.20**	**5.06 ± 0.11**
Un_26.96_433	1.00 ± 0.14	**4.25 ± 0.15**	**7.59 ± 0.14**
Un_27.2_217	1.00 ± 0.13	0.70 ± 0.21	**0.29 ± 0.05**
Un_28.39_446	1.00 ± 0.31	**5.78 ± 0.18**	**15.86 ± 0.16**
Un_28.68_446	1.00 ± 0.31	**5.54 ± 0.16**	**12.42 ± 0.13**
Un_30.54_217	1.00 ± 0.29	**11.51 ± 0.34**	**26.22 ± 0.06**
Un_30.72_217	1.00 ± 0.29	**11.70 ± 0.34**	**25.98 ± 0.06**
Un_33.11_433	1.00 ± 0.07	0.79 ± 0.16	**0.19 ± 0.14**

Data represents mean of 5 representatives of 10-pooled samples +/- s.e. Values that are highlighted in *green* (and bold) have a *t*-Test value $P < 0.05$ / (number of metabolites) while those with cyan (and bold) highlight are between this *P* value and 0.05 (i.e. below 0.05, but not below the Bonferroni-corrected *P* value).

Table S3: Response ratios of metabolites in root zones of Clipper and Sahara subjected to 100 mM salt-stress.

Metabolite	Region 1(CDZ)		Region 2 (EZ)		Region 3 (MZ)	
**Amino acids and amines**	**Clipper**	**Sahara**	**Clipper**	**Sahara**	**Clipper**	**Sahara**
Alanine	**1.83 ± 0.05**	1.05 ± 0.04	**1.83 ± 0.09**	**1.28 ± 0.05**	**1.56 ± 0.09**	**1.40 ± 0.08**
Allantoin	1.24 ± 0.10	1.20 ± 0.12	**1.40 ± 0.06**	0.85 ± 0.10	**1.89 ± 0.12**	**2.07 ± 0.12**
Arginine	1.47 ± 0.13	0.69 ± 0.17	**1.76 ± 0.08**	1.13 ± 0.15	**2.87 ± 0.23**	**1.62 ± 0.07**
Asparagine	**3.84 ± 0.12**	0.93 ± 0.20	2.78 ± 0.23	0.94 ± 0.14	**3.45 ± 0.24**	1.17 ± 0.07
Aspartate	**1.31 ± 0.06**	1.09 ± 0.06	1.58 ± 0.08	1.19 ± 0.08	**1.41 ± 0.05**	**1.27 ± 0.06**
Butyric acid,4-amino (GABA)	1.89 ± 0.27	0.83 ± 0.18	1.65 ± 0.16	1.34 ± 0.17	1.26 ± 0.14	0.94 ± 0.09
Cysteine	1.08 ± 0.15	0.85 ± 0.12	**1.60 ± 0.13**	0.75 ± 0.10	2.43 ±0.34	1.08 ± 0.04
Glutamate	1.09 ± 0.04	1.02 ± 0.07	**1.42 ± 0.09**	1.20 ± 0.09	**1.48 ± 0.05**	**1.47 ± 0.08**
Glutamine	**1.72 ± 0.12**	0.87 ± 0.13	1.40 ± 0.21	0.71 ± 0.14	1.30 ± 0.13	1.36 ± 0.19
Glycine	1.15 ± 0.03	**0.81 ± 0.07**	1.36 ± 0.05	0.89 ± 0.05	1.09 ± 0.07	1.22 ± 0.03
Guanine	**0.35 ± 0.05**	1.63 ± 0.26	1.39 ± 0.11	0.57 ± 0.10	1.00 ± 0.12	1.15 ± 0.23
Histidine	**1.82 ± 0.13**	0.78 ± 0.45	1.24 ± 0.09	0.85 ± 0.13	1.62 ± 0.17	1.26 ± 0.06
Homoserine	1.12 ± 0.17	0.87 ± 0.19	1.18 ± 0.08	1.18 ± 0.05	**1.79 ± 0.07**	1.22 ± 0.10
Isoleucine	1.31 ± 0.03	0.90 ± 0.11	1.18 ± 0.07	0.97 ± 0.06	**1.18 ± 0.05**	1.05 ± 0.03
Leucine	1.24 ± 0.02	0.81 ± 0.10	1.16 ± 0.06	1.03 ± 0.05	1.07 ± 0.04	1.07 ± 0.03
Methionine	0.98 ± 0.05	1.16 ± 0.29	1.16 ± 0.09	**0.66 ± 0.08**	0.92 ± 0.06	1.02 ± 0.02
N-acetyl serine	1.46 ± 0.11	1.37 ± 0.13	1.45 ± 0.13	0.23 ± 0.15	1.65 ± 0.14	1.30 ± 0.23
Ornithine	1.69 ± 0.24	0.92 ± 0.19	**1.62 ± 0.10**	1.18 ± 0.12	**2.67 ± 0.16**	**1.71 ± 0.07**
Phenylalanine	1.02 ± 0.04	1.15 ± 0.14	1.08 ± 0.10	0.76 ± 0.07	0.99 ± 0.05	**0.83 ± 0.05**
Proline	**5.67 ± 0.04**	**5.35 ± 0.09**	**2.25 ± 0.07**	**2.27 ± 0.05**	**1.69 ± 0.06**	**1.55 ± 0.06**
Putrescine	1.35 ± 0.34	0.94 ± 0.13	**0.64 ± 0.09**	**0.47 ± 0.18**	0.70 ± 0.26	0.75 ± 0.07
Pyroglutamate	**1.27 ± 0.04**	0.92 ± 0.08	**1.33 ± 0.06**	0.87 ± 0.08	1.06 ± 0.08	1.14 ± 0.09
Serine	**1.56 ± 0.03**	1.19 ± 0.10	1.40 ± 0.03	1.03 ± 0.06	**1.23 ± 0.03**	**1.22 ± 0.04**
Threonine	**2.29 ± 0.06**	1.92 ± 0.30	**2.76 ± 0.10**	**2.14 ± 0.04**	1.41 ± 0.06	1.07 ± 0.11
Tryptophan	0.74 ± 0.58	1.71 ± 0.25	0.96 ± 0.22	0.78 ± 0.21	1.35 ± 0.20	1.10 ± 0.09
Tyramine	0.69 ± 0.08	**0.25 ± 0.12**	0.98 ± 0.05	0.72 ± 0.08	0.90 ± 0.06	0.93 ± 0.05
Tyrosine	1.15 ± 0.03	0.97 ± 0.06	**1.45 ± 0.08**	0.87 ± 0.07	0.92 ±0.06	0.84 ± 0.07
Valine	**2.69 ± 0.04**	**1.47 ± 0.07**	**1.62 ± 0.04**	1.21 ± 0.03	**1.17 ± 0.04**	1.01 ± 0.07
β Alanine	**2.67 ± 0.10**	1.10 ± 0.10	**2.09 ± 0.02**	1.54 ± 0.06	**1.53 ± 0.09**	**1.49 ± 0.06**

**Organic acids**						
alpha-Ketoglutarate	0.65 ± 0 .27	0.50 ± 0.31	0.96 ± 0.07	**0.60 ± 0.07**	1.17 ± 0.06	1.57 ± 0.08
Citrate	0.77 ± 0.08	0.96 ± 0.32	1.51 ± 0.16	1.07 ± 0.11	**2.07 ± 0.15**	1.36 ± 0.05
Fumarate	**0.61 ± 0.05**	**0.51 ± 0.05**	1.42 ± 0.11	1.21 ± 0.12	1.37 ± 0.14	1.21 ± 0.19
Glucarate	0.51 ± 0.07	1.00 ± 0.24	1.07 ± 0.10	0.67 ± 0.32	1.57 ± 0.08	1.09 ± 0.07
Gluconic acid	0.73 ± 0.17	0.64 ± 0.23	**5.90 ± 0.25**	0.66 ± 0.33	2.36 ± 0.28	**2.51 ± 0.06**
Glycerate	**0.78 ± 0.07**	0.87 ± 0.12	1.01 ± 0.08	**0.61 ± 0.07**	0.99 ± 0.11	0.90 ± 0.10
Glycolate	1.12 ± 0.13	1.06 ± 0.19	0.93 ± 0.21	0.88 ± 0.11	1.09 ± 0.19	1.41 ± 0.16
Malate	**0.53 ± 0.03**	**0.46 ± 0.08**	0.87 ± 0.08	**0.58 ± 0.11**	0.82 ± 0.08	0.93 ± 0.14
Oxalate	0.71 ± 0.12	0.91 ± 0.06	1.13 ± 0.10	0.92 ± 0.07	1.02 ± 0.08	0.85 ± 0.08
Pipecolate	**2.34 ± 0.13**	**1.86 ± 0.09**	1.95 ± 0.28	**2.32 ± 0.19**	**1.67 ± 0.14**	**1.83 ± 0.10**
Quinate	0.55 ± 0.14	0.70 ± 0.17	1.22 ± 0.30	**0.46 ± 0.14**	0.55 ± 0.14	0.60 ± 0.01
Shikimate	0.63 ± 0.31	0.54 ± 0.50	3.77 ± 0.66	**0.10 ± 0.15**	0.87 ± 0.22	**3.88 ± 0.27**
Succinate	**0.78 ± 0.03**	0.80 ± 0.11	1.10 ± 0.08	0.79 ± 0.13	**1.29 ± 0.07**	0.94 ± 0.11
Threonate	0.86 ± 0.07	0.91 ± 0.13	1.50 ± 0.07	1.13 ± 0.07	1.26 ± 0.14	0.89 ± 0.14
**Fatty Acids**						
Palmitic Acid (Hexadecanoate)	0.91 ± 0.04	**1.51 ± 0.06**	1.28 ± 0.11	0.65 ± 0.10	1.21 ± 0.11	1.27 ± 0.14
Adipic Acid (Hexanedioic acid)	1.20 ± 0.10	0.85 ± 0.10	1.37 ± 0.09	1.11 ± 0.06	**1.77 ± 0.05**	1.04 ± 0.04
Stearic Acid (Octadecanoate)	0.87 ± 0.06	1.40 ± 0.10	1.38 ± 0.15	0.88 ± 0.15	1.70 ± 0.26	1.62 ± 0.18
Linoleic acid	1.13 ± 0.19	0.81 ± 0.15	1.37 ± 0.26	0.82 ± 0.17	0.89 ± 0.13	1.33 ± 0.33
Octadecanol	0.94 ± 0.14	**1.69 ± 0.09**	1.41 ± 0.30	1.08 ± 0.09	1.62 ± 0.25	1.38 ± 0.13
Oleic acid	0.72 ± 0.08	0.70 ± 0.17	1.33 ± 0.28	0.73 ± 0.17	0.99 ± 0.14	1.08 ± 0.39
**Sugars and Sugar Phosphates**						
Erythrose	1.27 ± 0.23	1.11 ± 0.28	0.95 ± 0.35	0.52 ± 0.33	**2.24 ± 0.21**	0.57 ± 0.07
Fructose	0.61 ± 0.24	0.55 ± 0.20	1.03 ± 0.12	0.45 ± 0.16	0.96 ± 0.21	1.04 ± 0.18
Fructose_6_Phosphate	0.47 ± 0.08	**0.28 ± 0.08**	1.24 ± 0.12	1.00 ± 0.11	1.16 ± 0.21	0.75 ± 0.06
Galactinol	1.11 ±0.16	0.81 ± 0.14	**2.06 ± 0.18**	**1.69 ± 0.08**	**1.36 ± 0.07**	1.17 ± 0.14
Galactonate	0.66 ±0.28	0.62 ± 0.43	1.31 ± 0.21	0.82 ± 0.23	0.97 ± 0.10	1.41 ± 0.10
Gluconate-6-phosphate	1.45 ± 0.26	1.03 ± 0.17	1.02 ± 0.18	1.42 ± 0.17	1.44 ± 0.26	1.51 ± 0.21
Glucose	0.56 ± 0.27	**0.51 ± 0.15**	1.12 ± 0.16	0.76 ± 0.13	1.01 ± 0.19	1.16 ± 0.18
Glucose-6-phosphate	0.41 ± 0.03	**0.26 ± 0.09**	1.00 ± 0.09	0.84 ± 0.09	1.09 ± 0.16	1.14 ± 0.27
Glycerol	**0.32 ± 0.05**	1.34 ± 0.23	0.92 ± 0.30	**0.27 ± 0.10**	0.86 ± 0.13	1.55 ± 0.15
Glycerol-3-phosphate	1.29 ± 0.09	1.09 ± 0.18	1.72 ± 0.33	1.59 ± 0.21	1.46 ± 0.25	0.94 ± 0.05

Glycerophosphoglycerol	0.54 ± 0.06	**0.57 ± 0.05**	0.74 ± 0.09	1.67 ± 0.22	**2.49 ± 0.35**	2.36 ± 0.30
Inositol	1.00 ± 0.12	0.90 ± 0.12	1.22 ± 0.18	1.09 ± 0.15	0.93 ± 0.15	1.25 ± 0.16
Inositol-1-phosphate	0.77 ± 0.07	0.85 ± 0.21	0.77 ± 0.05	1.09 ± 0.16	0.94 ± 0.19	**0.83 ± 0.04**
Maltose	1.49 ± 0.09	0.96 ± 0.22	**1.87 ± 0.05**	1.14 ± 0.16	0.85 ± 0.03	0.99 ± 0.03
Raffinose	**0.43 ± 0.24**	**0.30 ± 0.17**	1.48 ± 0.15	0.94 ± 0.14	**1.83 ± 0.10**	**1.60 ± 0.11**
Ribonate	0.70 ± 0.34	6.74 ± 0.55	0.78 ± 0.26	0.86 ± 0.18	1.35 ± 0.24	1.38 ± 0.33
Sucrose	1.56 ± 0.14	0.78 ± 0.11	**1.74 ± 0.13**	1.24 ± 0.12	1.95 ± 0.24	1.53 ± 0.15
Trehalose	0.58 ± 0.08	0.96 ± 0.23	1.09 ± 0.10	0.97 ± 0.13	0.93 ± 0.10	1.45 ± 0.29
Xylose	0.73 ± 0.33	1.38 ± 0.18	2.21 ± 0.35	0.89 ± 0.24	0.97 ± 0.35	**3.19 ± 0.09**
**Other compounds**						
Adenosine	1.37 ± 0.14	1.14 ± 0.29	0.87 ± 0.22	0.86 ± 0.11	0.86 ± 0.30	0.63 ± 0.18
Campersterol	**1.70 ± 0.09**	1.03 ± 0.05	0.83 ± 0.16	1.08 ± 0.10	1.20 ± 0.13	1.90 ± 0.38
Ethanolamine	0.98 ± 0.03	**0.69 ± 0.12**	1.01 ± 0.15	0.93 ± 0.04	1.22 ± 0.13	1.33 ± 0.16
Fucosterol	1.00 ± 0.11	**0.51 ± 0.12**	0.52 ± 0.21	0.86 ± 0.13	1.30 ± 0.12	0.92 ± 0.08
Indole-3-acetate (IAA)	2.21 ± 0.23	0.65 ± 0.16	2.13 ± 0.39	0.91 ± 0.29	1.52 ± 0.26	0.88 ± 0.14
Monomethylphosphate	0.67 ± 0.19	**1.67 ± 0.08**	1.31 ± 0.24	1.24 ± 0.11	1.28 ± 0.14	1.28 ± 0.16
Phosphate	0.87 ± 0.06	1.22 ± 0.15	1.13 ± 0.18	1.21 ± 0.10	1.05 ± 0.06	**2.15 ± 0.19**
Uracil	1.23 ± 0.08	**0.37 ± 0.12**	1.16 ± 0.07	**0.61 ± 0.12**	1.44 ± 0.13	**0.76 ± 0.05**
**Unknowns**						
Un_11.54_417	0.85 ± 0.04	1.35 ± 0.09	1.32 ± 0.14	0.95 ± 0.12	1.02 ± 0.14	1.18 ± 0.19
Un_12.91_273	0.83 ± 0.09	**0.48 ± 0.05**	1.31 ± 0.08	0.73 ± 0.09	1.04 ± 0.15	1.08 ± 0.22
Un_13.96_284	0.91 ± 0.06	**1.86 ± 0.21**	0.93 ± 0.14	**0.65 ± 0.07**	1.28 ± 0.12	1.39 ± 0.13
Un_14.37_158	0.85 ± 0.09	**0.24 ± 0.15**	1.22 ± 0.11	0.83 ± 0.11	1.18 ± 0.12	0.89 ± 0.13
Un_14.87_191	1.47 ± 0.09	1.46 ± 0.08	1.70 ± 0.40	0.94 ± 0.15	1.20 ± 0.18	1.20 ± 0.12
Un_15.01_115	**3.66 ± 0.19**	0.70 ± 0.13	**2.75 ± 0.21**	1.00 ± 0.15	**2.39 ± 0.05**	1.15 ± 0.11
Un_15.07_261	**2.67 ± 0.15**	1.31 ± 0.24	**2.63 ± 0.17**	1.10 ± 0.14	2.92 ± 0.34	0.98 ± 0.08
Un_16.77_591	**0.76 ± 0.05**	0.84 ± 0.07	1.12 ± 0.10	1.02 ± 0.11	**1.45 ± 0.05**	**1.60 ± 0.10**
Un_17.26_102	0.63 ± 0.15	**0.38 ± 0.26**	1.04 ± 0.41	0.74 ± 0.12	0.98 ± 0.34	0.59 ± 0.11
Un_18.1_345	1.51 ± 0.17	0.77 ± 0.20	1.20 ± 0.07	0.93 ± 0.08	0.80 ± 0.15	1.05 ± 0.10
Un_18.11_116	0.76 ± 0.16	**0.22 ± 0.21**	1.68 ± 0.30	1.04 ± 0.09	0.96 ± 0.13	0.79 ± 0.08
Un_18.4_242	1.19 ± 0.08	**1.66 ± 0.10**	1.08 ± 0.10	0.82 ± 0.14	1.93 ± 0.29	2.13 ± 0.33
Un_19.41_156	1.83 ± 0.13	0.73 ± 0.13	1.71 ± 0.18	0.83 ± 0.10	1.51 ± 0.19	1.01 ± 0.09
Un_22.25_217	1.01 ± 0.12	0.85 ± 0.21	1.39 ± 0.16	0.39 ± 0.18	1.31 ± 0.22	1.57 ± 0.24

Un_25.61_379	1.08 ± 0.11	**0.48 ± 0.12**	1.00 ± 0.09	0.72 ± 0.31	0.93 ± 0.31	**1.63 ± 0.15**
Un_26.96_433	1.04 ± 0.16	**0.61 ± 0.15**	1.08 ± 0.08	0.79 ± 0.26	0.71 ± 0.21	0.62 ± 0.24
Un_27.2_217	0.79 ± 0.06	1.20 ± 0.15	0.89 ± 0.05	1.35 ± 0.14	1.09 ± 0.06	1.24 ± 0.10
Un_28.39_446	1.10 ± 0.12	**0.41 ± 0.13**	1.14 ± 0.09	0.65 ± 0.34	0.66 ± 0.25	0.87 ± 0.25
Un_28.68_446	1.04 ± 0.11	0.45 ± 0.11	1.20 ± 0.05	1.04 ± 0.32	0.75 ± 0.23	0.92 ± 0.20
Un_30.54_217	0.54 ± 0.05	0.74 ± 0.12	**0.51 ± 0.05**	0.78 ± 0.11	0.97 ± 0.09	1.45 ± 0.27
Un_30.72_217	0.54 ± 0.05	0.74 ± 0.12	**0.48 ± 0.05**	0.73 ± 0.11	0.98 ± 0.10	1.46 ± 0.27
Un_33.11_433	0.72 ± 0.08	1.04 ± 0.25	1.22 ± 0.10	1.32 ± 0.11	**1.43 ± 0.07**	1.80 ± 0.30

Data represents mean of 4-5 representatives of 10-pooled samples +/- s.e. Values that are highlighted in *green* (and bold) have a *t*-Test value $P < 0.05$ / (number of metabolites) while those with cyan (and bold) highlight are between this P value and 0.05 (i.e. below 0.05, but not below the Bonferroni-corrected P value).
